# Critical Factors Influencing Decision to Adopt Human Resource Information System (HRIS) in Hospitals

**DOI:** 10.1371/journal.pone.0160366

**Published:** 2016-08-05

**Authors:** Md Golam Rabiul Alam, Abdul Kadar Muhammad Masum, Loo-See Beh, Choong Seon Hong

**Affiliations:** 1 Department of Computer Science and Engineering, Kyung Hee University, Yongin-si, Korea; 2 Department of Administrative Studies & Politics, University of Malaya, Kuala Lumpur, Malaysia; Southwest University, CHINA

## Abstract

The aim of this research is to explore factors influencing the management decisions to adopt human resource information system (HRIS) in the hospital industry of Bangladesh—an emerging developing country. To understand this issue, this paper integrates two prominent adoption theories—Human-Organization-Technology fit (HOT-fit) model and Technology-Organization-Environment (TOE) framework. Thirteen factors under four dimensions were investigated to explore their influence on HRIS adoption decisions in hospitals. Employing non-probability sampling method, a total of 550 copies of structured questionnaires were distributed among HR executives of 92 private hospitals in Bangladesh. Among the respondents, usable questionnaires were 383 that suggesting a valid response rate of 69.63%. We classify the sample into 3 core groups based on the HRIS initial implementation, namely adopters, prospectors, and laggards. The obtained results specify 5 most critical factors i.e. IT infrastructure, top management support, IT capabilities of staff, perceived cost, and competitive pressure. Moreover, the most significant dimension is technological dimension followed by organisational, human, and environmental among the proposed 4 dimensions. Lastly, the study found existence of significant differences in all factors across different adopting groups. The study results also expose constructive proposals to researchers, hospitals, and the government to enhance the likelihood of adopting HRIS. The present study has important implications in understanding HRIS implementation in developing countries.

## Introduction

To achieve organisational goal, traditional human resource management (HRM) processes have been shifted to strategic HRM through a significant contribution of Information Technology (IT) [1]. Now, this IT backed HRM is renamed as human resource information system (HRIS) [2]. Some organisations are busy intensifying of HRIS, while other organisations have failed to realize its short-term and long-term benefits given the misperception about HRIS and lack of managerial foresightedness. Realizing the magnitude of HRIS applications, researchers explored a broad array of influential factors for adoption decision and implementation of HRIS among business organisations [3]. The preceding studies indicate that only the firm size is consistently the accepted factor among the probable factors for HRIS adoption [1]. However, researchers argued that the weight of explored factors and the relative weight of every variable may be changed along with innovation characteristics and its setting [4, 5]. Moreover, scholars revealed that the results of technology innovation research are inconsistent [6]. Hence, for a particular setting, it is essential to recognize the potential factors that influence decision of HRIS adoption in the organisations.

Generally, major amount of studies on HRIS have been focusing on developed countries, such as, the US, Canada, and Western Europe [7], while scarce in developing countries [8]. Besides, there is an acute shortage of HRIS research with special focus on Bangladesh. Although Bangladesh has been considered as a “Next Eleven” emerging economy, its HRIS progress is in the early stage [9]. Research has argued that most of the organisations in Bangladesh are not aware of HRIS despite its multitudinous convenience. But, the trend is changing gradually and organisations are adopting information system (IS) for their daily business processes. However, the results of these efforts have not been very noticeable in the healthcare sector of Bangladesh owing to the lack of a clear vision, policy, and strategy. Recently, some technical supports were received from the world health organisation (WHO) for the assessment of the present healthcare system and development of a wide-ranging human resource information system (HRIS) in Bangladesh.

The concept of adoption of technology innovation has been considered universal [4]. But, there are certain constrains regarding the suitability of adoption of Western innovation models when these are to be adopted in non-Western countries [8]. For instance, being a developing Asian country, Bangladesh is evidently different from the Western societies in the context of technology, economy, and environment. So, exploring the applicability of HRIS adoption model in Bangladesh is a crying need.

For any innovation adoption, Rogers [10] categorised the organisations into adopters, prospectors, and laggards, the extent to which an organisation comparatively adopt innovation than others. First, organisations that have already implemented technological innovation for their business processes are categorised as adopters. Second, those organisations that have not yet employed the technological innovation, but have an explicit plan to adopt and implement innovation in the near future are categorized as prospectors. Usually, they willingly accept innovation that has been proven effective. The last category is the laggards where the technological innovation is neither implemented nor planned to adopt innovation in immediate future. Although these organisations may agree on the adoption of the state-of-the-art technological innovation being compelled by industry pressure, but they are naturally relaxed in adopting technological innovation. In respect to the status of HRIS innovativeness adoption [10], we also categorize hospitals into 3 main groups.

The prime objective of this paper is in finding the most significant factors that are linked to managerial decision for adapting HRIS in the hospital sector of Bangladesh. The following section comprises the literature review. By extensive review process, the variables used in our analysis were selected. The research methodology section is presented a research model of HRIS adoption, variables definition and their measurement methods. The details of data analysis and the outcomes of the study are deliberated in data analysis and results section. A brief description on results is stated in discussion section. The recommendations and future research directions are discussed in conclusion section.

## Literature Review

The success of any national healthcare system depends on efficient and effective HRM. So, a country needs accurate statistical information on human resource for health (HRH) to ensure that the right personnel are in the right place with the right skills. But, there is lack of accessibility of HRH specific information in developing countries [11]. A robust HRIS can help administrators and policy makers to quickly respond to HR related questions affecting healthcare service delivery. In spite of tremendous advantages of information systems (ISs), the wide adoption of ISs in healthcare organization is not very common in developing countries. Sometimes, these systems are not accepted very well by their users in hospitals [12]. There are many critical factors which direct the adoption of ISs and influence the acceptance of such renovation in healthcare organizations. These factors have been studied in several researches persuading the decision to adopt HRIS in an organisation [1, 13–15].

Researchers explored some imperative factors related to technology innovation adoption. In this paper, we scrutinize some factors for adopting HRIS taken from IS/IT innovation literature. Both qualitative and quantitative approaches are employed in technology adoption research (see Table 1). However, the qualitative is a comprehensively noticeable approach. It is evident from reviewing previous studies that, the influential factors are divided into four dimensions e.g. human, organisation, technology, and environment.

**Table 1:** Summary of IS adoption literature.

Dimension & Factors/Authors	[58]	[38]	[80]	[38]	[49]	[1]	[13]	[32]	[22]	[77]	[81]	[82]	[83]	[57]	[84]	[36]	[85]	[86]	[87]	[88]	[89]	[70]	[76]	[68]
**Human**																								
Innovativeness of senior executives		√		√			√		√															√
IT capabilities of staff	√			√	√	√	√	√	√	√	√	√	√	√		√				√	√		√	
**Technological**																								
IT Infrastructure	√		√	√	√			√		√		√	√		√	√	√	√				√	√	
Perceived compatibility	√	√	√	√	√	√	√	√		√	√		√		√				√			√		
Perceived complexity	√	√	√	√		√	√	√	√	√	√		√				√							
**Organisational**																								
Relative advantage		√		√		√	√	√	√	√	√	√	√								√	√		√
Centralisation		√	√	√				√						√		√						√		
Formalisation		√		√				√								√						√		
Top management support	√			√	√	√	√	√		√	√		√	√			√		√			√	√	
Perceived cost							√			√				√	√		√					√		√
**Environmental**																								
Competitive pressure	√		√	√		√		√		√	√	√			√	√	√		√	√	√	√	√	√
Technology vendor support		√		√	√		√	√		√	√			√								√		√
Government regulations and support				√	√		√	√		√				√						√	√	√	√	

The human factors include the hospital senior executives and employees with IS competence [16]. Several researchers reported that senior executives such as Chief Information Officer (CIO), Chief Executive Officer (CEO), and other senior executives play a vital role in usage of ISs at organisational level [13]. Senior executives’ direct involvement in IS activities is not only an indication of the significance of IS, but also guarantee their support and cooperation for the overall success of the IS initiatives in the organisation [17]. A contemporary study by Al-Qirim [18]revealed that electronic commerce adoption in New Zealand is positively affected by CEO innovativeness. Consistent with prior studies, a study on small and medium-sized enterprises (SMEs) claimed that owner’s IT ability and innovativeness were significant factors to adopting electronic commerce applications in Indonesia [19]. Researchers argued that personnel’s IT capabilities should be considered in the process of adopting essential IS applications in the hospital setting [20, 21]. According to Hung, Hung et al. [22], the information intensity and the IS knowledge of employees are important predictors of IS application adoption in the hospitals. Moreover, Ettlie [23] argued that employees must hold some IS knowledge in order to utilize new implemented IS more competently. Therefore, if the employees have adequate knowledge and skills on newly adopted IT applications, hospitals will undoubtedly posit more confidence for the whole adoption process [13]. From the above literature review, following hypotheses were suggested for the adoption of HRIS in hospitals.

H1: The 3 categories of hospital significantly vary in the extent of Innovativeness of senior executives.

H2: The 3 categories of hospital significantly vary in the extent of IT capabilities of staff.

The technological dimension refers to the technical issues involved in HRIS technology adoption [16]. IT infrastructure, perceived complexity, and perceived compatibility are reported as significant factors under this dimension. Among the IT adoption factors, robust IT infrastructure is the most essential factor to adopt a new IS application [24]. According to Zhu, Kraemer et al. [25], IT infrastructure is less advanced in most organisations of developing countries. For instance, most of organisations in Pakistan usually encounter different types of obstacle in implementation of suitable software and hardware due to weak IT infrastructure [26]. In addition, researcher argued that owing to lack of knowledge and skills, the perceived complexity of new technology adoption leads to resistance [27]. Generally, perceived system complexity and compatibility are key criteria when making the decision of adoption [20, 21]. The state-of-the-art information systems, which are unique in nature, are used in hospitals. Among the state-of-the-arts the Radiological Information System (RIS), the Hospital Information System (HIS), and the Picture Archiving and Communication System (PACS) are mentionable information systems [16]. Moreover, earlier research specified that perceived complexity and perceived compatibility influence the managerial decision of adoption of up to date information system positively [28]. So, organisations ought to consider how these information systems can work together. Therefore, the level of system complexity and compatibility are important criteria when making an adoption decision of HRIS. Hence, we proposed following three hypotheses for the adoption of HRIS.

H3: The 3 categories of hospital significantly vary in the presence of IT infrastructure.

H4: The 3 categories of hospital significantly vary in the extent of perceived complexity.

H5: The 3 categories of hospital significantly vary in the extent of perceived compatibility.

Organisational factors, includes the structure and process of an organisation, can constrain or facilitate the adoption or implementation of technological innovations [29]. The most commonly surveyed attributes in IT adoption in organisations were the factors of organisational dimension [30]. In previous studies, relative advantage, centralisation, formalisation, top management support, and perceived cost were identified as significant organisational factors that affect any organisation’s intention of adopting modern technologies in the information systems (Table 1). The relative advantage is the belief of certain benefits in terms of economic profitability, costs reduction, performance improvement by savings in time and effort or in other ways [31]. Teo, Lim et al. [1] pointed out that the perceived advantages of HRIS for HR department has a very progressive relationship with the decision to adopt the system in Singaporean companies. With that same train of thought, Al-Dmour [32] explored that the perceived relative advantage is a significant factor for HRIS adoption in Jordanian firms. Again, the willingness or intention of the top-level management is called as top managerial level support of the organisations to make available the essential resources and authority or power for the success of IT/IS project [13]. A study on information system adoption in hospitals stated that top manager’s support affects a new technology adoption [33]. In the same vein, Teo, Lim et al. [1] found top management support as a significant factor among organisational factors for adoption and diffusion of HRIS in Singapore. Another important factor is centralisation. Centralised organisational structures depend on few persons such as top-level decision makers to make decisions and provide direction for the company [27]. Numerous research specified that the more centralised design in an organization, the more likely to adopt strategic IS [34], which leads the end-user computation becoming more effective [32]. With that same reasoning, Zmud [35] stated that centralisation is related positively with the initiation, adoption and implementation of incompatible technical innovations. Another factor, formalisation denotes the level of belief on formal procedures, regulations, and task boards of an organisation for smooth management of organisational activities and work flows [36]. Dembla, Palvia et al. [37] revealed that formalisation is an important factor of technology innovation. In support of this proposition, England and Stewart [38] reported that both health and banking sectors of Australia show high levels of formalisation. They also identified formalisation as an imperative factor for any technological innovation in Australia. Finally, the cost, directly or indirectly related to adoption, is a major factor in the adoption and utilization of any new technology. When an organisation makes a decision on whether to adopt a new technology, a cost benefit analysis is performed for its feasibility [39]. With the same chain of thought, Premkumar and Roberts [40] attested that the less expensive the cost of a certain innovation the more probable it will be implemented in the organisations. Also, innovation cost is expected to negatively affect innovation adoption [41]. The cost for technology innovation includes the potential administrative and implementation costs. Moreover, cost related to IS adoption includes operating costs, setup costs, and training costs [42]. Thus, we can propose that:

H6: The 3 categories of hospital significantly vary in the extent of relative advantage (perceived benefits).

H7: The 3 categories of hospital significantly vary in the extent of top management support of HRIS.

H8: The 3 categories of hospital significantly vary in the extent of centralisation approach in the organisation.

H9: The 3 categories of hospital significantly vary in the extent of formalisation approach in the organisation.

H10: The 3 categories of hospital significantly vary in the extent of perceived cost for HRIS.

The last dimension—environment—denotes the external factors of IS adoption in hospital sector. The environmental dimension comprises the competitive pressure, technology vendor support, and government regulations and support [43]. Among the variables, competitive pressure denotes the degree of pressure that a company faces from competitors within the industry [44]. Competitive pressure forces organisations to become pioneer in adoption of the state-of-the-art IS applications for gaining strategic benefits over the competitors and also for delivering quality services [14, 45, 46]. A study on Taiwan conducted by Hsiao, Li et al. [47] attested that competitive pressure significantly influences hospital management to adopt mobile nursing systems. Technology vendor refers to suppliers of IT-related goods and services to other companies [28]. Similarly, Costa, de Oliveira et al. [48]and Sulaiman and Wickramasinghe [49] also revealed that technology vendor support has statistically significant influence on adoption of IS innovation. Last but not least, government regulations and support is an important factor in the environmental context. Numerous researchers revealed that government regulations and support has a significant influence on adoption decision of IT innovation in developing countries [13, 33]. In the same vein, some research on developing countries shows the empirical evidence that government regulations and polices positively effects the decision to adopt IT adoption in healthcare sector [16, 28, 32, 47, 49]. This discussion leads to the following hypotheses:

H11: The 3 categories of hospital significantly vary in the extent of competitive pressure for adopting HRIS.

H12: The 3 categories of hospital significantly vary in the extent of technology vendor support for HRIS.

H13: The 3 categories of hospital significantly vary in the extent of government regulations and support for HRIS.

In Bangladesh, the healthcare system comprises of both public and private sectors. The public hospitals are run by the central government. These hospitals provide services at subsidized rates or free of cost to low-income groups in rural and urban areas. These hospitals are not accustomed with IS applications. The private hospitals include specialist hospitals, corporate hospitals and small hospitals. And, these are expensive for healthcare service. In some private hospitals, IS applications are used by administrative personnel, medical personnel, customer service personnel, information personnel for their daily business process, and these systems affect employees’ job satisfaction and even job performance. Consequently, the organisational performance is affected. Bangladesh is suffering from acute shortage of health workers. Moreover, the complete picture of human resources for health (HRH) in Bangladesh or a comprehensive statistics on HRH is absent for both informal and formal health sectors. Those data are very crucial and can play important roles in developing an effective HRH policy for citizen’s shifting towards their healthcare needs [50]. So, adoption of HRIS will help the health sector to manage this shortage of workforce efficiently and effectively. The present study includes private hospitals of Bangladesh as most of public organisations failed to realize the benefit of HRIS [9].

## Research Methodology

### Conceptual framework

Around the world, healthcare is a large and emergent sector that is undergoing major revolution through different type of IT tools [51, 52] and IS facilities. The nature of IS applications is the same for the business processes, but its necessity and usage differ from industry to industry or country setting. To perceived its need, we found in past literature that TOE framework developed by Tornatzky and Fleischer [43], and HOT-fit model proposed by Yusof, Papazafeiropoulou et al. [53] and Yusof, Kuljis et al. [54], are apposite models for the present study. We investigated some factors that influence the management decision for HRIS innovation.

The TOE framework [43]is an organisation level theory that has vital impact on the adoption decisions of innovation. It describes 3 diverse dimensions of an organisation’s context i.e. technology, organisation, and environment. It is the most widely applied framework to examine the influence of adoption factors for technology innovation in the organisations. Some studies of technology adoption were conducted in hospital setting and found the aptness of applying the TOE framework [13, 16, 33, 55, 56]. Moreover, in the contemporary research, TOE framework was used to explore the determinants of HRIS adoption in organisations [57, 58]. Hence, it shows the aptness of applying TOE framework in the present research.

With a focal attention on industrialization of health information system, Yusof, Kuljis et al. [54] and Yusof, Papazafeiropoulou et al. [53] conducted examinations to examine the system adoption in the hospitals. They revealed that the alignment of human capacities, organisational factors, and technological strenght is strategic approach in IT adoption as one of these dimension significantly affects IT investment in the organisations. Hence, the significance of human expertise is acknowledged in the organisation, and consequently, it influences the adoption of IT innovation. In addition, some researchers found the suitability of applying the HOT-fit model in the hospital setting [13, 16].

**Fig 1:**
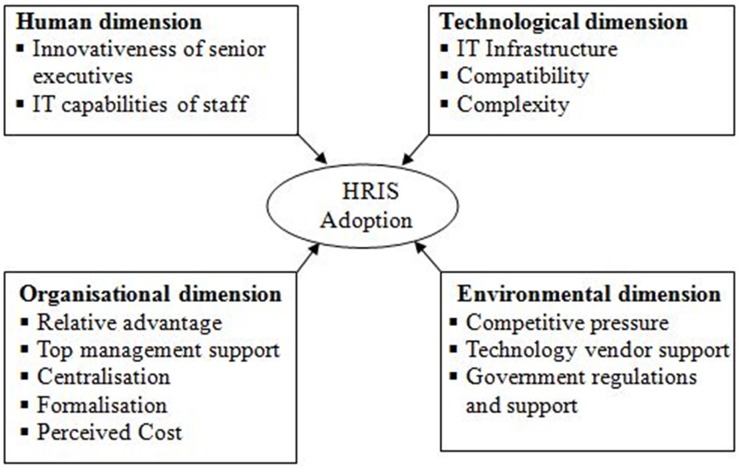
Conceptual Model. This is the conceptual framework of HRIS adoption model based on TOE framework and HOT-fit model.

Based on previous empirical research findings and theoretical background, this study develops the conceptual research framework (see Fig 1) by combining the HOT-fit model with TOE framework for HRIS adoption in healthcare sector. This conceptual model contains some selected factors under 4 dimensions of the technology adoption. This paper endeavors to offer helpful direction regarding hospital practitioners and decision-makers in improving and promoting a better decision in adopting HRIS technology in the hospital context of Bangladesh.

### Research instruments

The developed questionnaire was a closed question style. It has two parts. First part covers the questions on the basic information of the respondents and the hospital. Based on instrument of Rogers [10], the hospitals were asked if they were using HRIS by selecting which stage of HRIS adoption that they were in: 1) adopters–had already adopted HRIS applications, 2) prospectors—intended to adopt HRIS applications in the next three years, and 3) laggards—do not anticipate to adopt HRIS applications. In contemporary research, Lertwongsatien and Wongpinunwatana [59] and Alshamaila, Papagiannidis et al. [60] used this instrument to explore the determinants of technology innovation. The other part comprises the questions for identifying the current status of the HRIS in the hospital of Bangladesh, and the assumed 13 factors for adopting HRIS based on earlier technology adoption research. The responses were collected through a 7-point Likert scale, in which the lowest point ‘1’ represents “strongly disagree”, and highest point ‘7’ represents “strongly agree”, and middle point ‘4’ represents “neither agree nor disagree”. After the completion of the draft questionnaire, according to suggestion of Cooper and Schindler [61], three IS academics and three HRM academics with earned doctoral degree were requested to scrutinize and help modify the content of the questionnaire. To ensure the validity and reliability of the tentative scale, and also to evaluate impending complications in constructs uni-dimentionality, the five eminent IS and/or HRM senior executives from five hospitals were invited to scrutinize the questionnaire. Some amendments were suggested. The final questionnaire was developed with all possible suggestions included.

In this study, the only dependent variable is HRIS Adoption. There are 13 independent variables. “Innovativeness of senior executives” refers to willingness of senior executives to introduce innovation through testing innovative manners intended at developing new products, services. The construct is adapted from Thong and Yap [62], Agarwal and Prasad [63], Hung, Hung et al. [22]and Lian, Yen et al. [13]. It is measured via 4 items. “IT capabilities of staff” construct is assessed via 3 items scale adapted from Kuan and Chau [64], Teo, Lim et al. [1] and Hung, Hung et al. [22]. The construct refers to the degree that employees have IT knowledge and skill. “IT infrastructure” is measured using 4 items scale adapted from Grover [44], Hartono, Li et al. [65], Masum [14] and Al-Dmour [32]. The construct pertain to a common information technology platform containing soft-wares, hardwires, and communication and networking mediums and tools which are essential for transferring or executing business information. “Perceived compatibility” construct is measured via 3 items scale adapted from Teo, Lim et al. [1], Premkumar and Roberts [40], and Ainin, Salleh et al. [66]. It denotes the degree to which a technology adoption is perceived as being consistent with the current value, infrastructure, previous experiences, and essential of potential users or adopters. The construct—“Perceived complexity”—is measured via 4 items scale that represents the perceived comparative challenges to understand and use the innovation. These items are adopted from Chang, Hwang et al. [28], Premkumar and Roberts [40], Teo, Lim et al. [1]. The degree of advantage perceived by an individual user or organization for adopting innovation is referred to the “Relative advantage”. Measuring the construct is adapted from Moore and Benbasat [67], Ghobakhloo, Arias-Aranda et al. [68], Teo, Lim [1], Premkumar and Roberts [40], Hung, Hung et al. [22]. The construction of the “Top management support” is assessed through a scale of 4 items, which are adapted from McGinnis and Ackelsberg [69], Grover [44], Teo, Lim et al. [1] and Premkumar and Roberts [40]. The construct refers to the availability of a supportive environment and adequate resources provided by an organisation’s top management for adopting/ using HRIS in the organisation. “Centralisation” is measured using 4 items scale adapted from Zmud [35], Dembla, Palvia et al. [37] and Al-Somali, Gholami et al. [70]. The construct refers to the degree to which decision making power is given to the top level managers. “Formalisation” construct is assessed via 4 items scale adapted from Grover [44], Dembla, Palvia et al. [37] and Al-Somali, Gholami et al. [70]. The construct denotes the presence of written rules, procedures and documents for performing organisational activities. The construct—“Perceived cost”—is measured via 3 items scale that represents the implementation costs for technology innovations comprising initial development investments and recurring operating expenses for technology adoption. These items are adopted from Ghobakhloo, Arias-Aranda et al. [68], Kuan and Chau [64], Al-Somali, Gholami et al. [70], and Premkumar and Roberts [40]. “Competitive pressure” refers to the overall tendency, competition and direction of functioning practices that force an organisation to embrace new innovation so as to survive in the industry or bear its competitive advantages. This construct is adapted from Premkumar and Roberts [40], Teo, Lim et al. [1], Sophonthummapharn [71] and Masum [14]. The construct is measured via 3 items. “Technology vendor support” construct is assessed via 3 items scale adapted from Thong [72], Li, Chang et al. [73], Hsiao, Li et al. [47] and Ghobakhloo, Arias-Aranda et al. [68]. The construct refers to the degree to which vendors provide services such as vendors’ engagement in product installation to complete consultancy and supervision, and employee training. The last independent variable, “Government regulations and support” is measured using 2 items scale adapted from Kuan and Chau [64], Hsiao, Li et al. [47] and Al-Dmour [32]. The construct states the degree to which government initiates polices for sustenance and allocating various resources in implementation of HRIS.

### Participants and data collection

HRIS progress of Bangladesh is in early stage, and most public organisations failed to realize the multitudinous convenience of HRIS [9]. Some multinational companies and large private organisations are in better position compared to public sector [58]. So, the study includes 383 private hospitals (adopters, prospectors, and laggards) in Bangladesh. Employing non-probability sampling method, a total of 550 copies of structured questionnaires were distributed among HR managers or IS managers at different levels such as managers (mid-level managers) and senior executives (top level managers) of HRM and IT department. Among the respondents, 383 returned copies were valid, with a response rate of 69.64%. The data collection period was from January to April 2014.

### Ethical considerations

Ethical approval was provided by the Uttara University (UU), Bangladesh ethics committee (reference: UU/eq-c/799/10/2013), and all study works were performed in accordance with the national ethics regulations. Study participants were informed of the study purpose and of their right to keep information confidential. All participants provided their written consent to participate in this study.

## Data Analysis and Results

In this study, factor analysis was employed with varimax rotation to investigate the convergent and discriminant validity. To investigate the mean difference among the 3 groups (adopter, prospector and laggard), Analysis of Variances (ANOVA) was used. Subsequently, the mean differences of 3 hospital groups are compared in pair-wise fashion using the popular Scheffee’s method of post-hoc multiple comparison analysis.

### Respondent demographics

The usable questionnaires were collected from 383 large hospitals (where the number of HRIS adopters, prospectors, and laggards are 85, 162, and 136 respectively). The sample characteristics of respondents and hospitals are summarised in Table 2. The table demonstrates that 75.9% (291) of respondents have seniority (more than 5 years) in the healthcare sector of Bangladesh, 61.6% (236) of the respondents have more than 5 years’ job experience in their present position, and 37.9% (145) of respondents hold top level positions in a HRM or IS department. These results express that respondents were skilled to understand the questionnaires.

**Table 2:** Sample characteristics.

Respondent characteristic	Frequency	Percentage (%)
***Gender***		
Male	260	67.9
Female	123	32.1
***Age***		
26–31	29	7.6
31–39	124	32.3
39–45	147	38.4
45–58	83	21.7
***Education level***		
Bachelor’s	168	43.9
Master’s	189	49.3
M.Phil./PhD	26	6.8
***Roles of respondents***		
Senior Executive (HRM/IT)	145	37.9
Manager (HRM/IT)	238	62.1
***Seniority in current position***		
Above 15 years	16	4.1
10∼14 years	47	12.4
5∼9 years	173	45.1
1∼4 years	124	32.4
Less than 1 year	23	6.0
***Executives' seniority in the health care sector***		
Above 26 years	35	9.1
21∼25 years	57	14.9
16∼20 years	50	13.0
11∼15 years	74	19.3
6∼10 years	75	19.6
Less than 5 years	92	24.1
***HRIS adoption stage in hospitals***		
Adopters	85	22.2
Prospectors	162	42.3
Laggards	136	35.5

### Validity and reliability

The multi-item factors proposed in the model were analyzed to evaluate the reliability, and the validity of convergence and discrimination. To test the convergent validity and discriminant validity, factor analysis with varimax rotation was employed for 48 items. Most of the loadings value of each observed indicator on its latent construct exceeded 0.6 (threshold value), and the eigenvalue is larger than 1. So, good convergent validity was confirmed [74]. Due to cross low value and cross loading of the factor loading, 5 items were dropped. Finally, considering all of the 4 dimensions, the total 13 factors are extracted which includes 43 items. In Table 3, the variables definition and abbreviation are expressed as; V1: Innovativeness of senior executives; V2: IT capabilities of staff; V3: IT Infrastructure; V4: Perceived compatibility; V5: Perceived complexity; V6: Relative advantage; V7: Top management support; V8: Centralisation; V9: Formalisation; V10: Perceived cost; V11: Competitive pressure; V12: Technology vendor support; V13: Government regulations and support. The sample was suitable for the analyses as Kaiser–Meyer–Olkin (KMO) value was 0.72. The extracted factors cumulatively explained 83.14% of the variance. However, the Cronbach's alpha values were computed for construct reliability, and these were higher than 0.7 for each factor, which indicates sufficient reliability of the instrument [75].

**Table 3:** Construct reliability and validity analysis.

Dimensions	Items	Item description	Item loading	Cronbach's alpha	Significant of Bartlett’s test	Total variance explained (TVE)
**Human**	**Variable (V1): Innovativeness of senior executives**				0.00	59.71%
	Hitem1	Senior executives are enthusiastic to experiment a new information system.	0.79	0.79		
	Hitem2	Senior executives do not timid to try out new information systems.	0.71			
	Hitem3	Senior executives would sooner create something new than improve something existing.	0.89			
	Hitem4	Senior executives often risk doing things differently.	0.85			
	**Variable (V2): IT capabilities of staff**					
	Hitem5	All human resources personnel have IT skill to support human resources functions.	0.87	0.83		
	Hitem6	All human resources personnel are computer-literate.	0.66			
	Hitem7	There is at least one computer expert in the human resources department.	0.81			
**Technology**	**Variable (V3): IT Infrastructure**				0.00	66.76%
	Titem1	Our organisation is highly computerized with internal and external network connections that connect the firm with its branches.	0.78	0.90		
	Titem2	The organisation has sufficient software and database resources to support HRIS.	0.91			
	Titem3	The organisation has speedy internet facility.	0.77			
	Titem4	The organisation has a strong backup plan for network failure.	0.81			
	**Variable (V4): Perceived compatibility**					
	Titem5	Adoption of HRIS applications is compatible with existing operating practices.	0.89	0.87		
	Titem6	HRIS applications are consistent with our organisation’s values and belief.	0.95			
	Titem7	HRIS is/will be incompatible with existing hardware and network facilities.	0.76			
	Titem8	The implementation of the system is/will be incompatible with existing software applications and database system.	0.88			
	**Variable (V5): Perceived complexity**					
	Titem9	HRIS is complex to use.	0.68	0.93		
	Titem10	HRIS development is a complex process.	0.90			
	Titem11	HRIS is hard to learn.	0.92			
	Titem12	Integrating HRIS into our current work practices will be very difficult.	0.86			
**Organisation**	**Variable (V6): Relative advantages**				0.00	76.12%
	Oitem1	Using the HRIS will enhance my effectiveness on the job.	0.87	0.73		
	Oitem2	HRIS will allow us to enhance our productivity.	0.90			
	Oitem4	HRIS will allow us to cut costs in our operations.	0.93			
	Oitem6	Implementing of HRIS will increase organisation profitability.	0.79			
	**Variable (V7): Top management support**					
	Oitem7	Top management enthusiastically supports the adoption of HRIS.	0.83	0.82		
	Oitem9	Top management has allocated adequate resources for the adoption of HRIS.	0.88			
	Oitem10	Top management is aware of the benefits of HRIS.	0.73			
	**Variable (V8): Centralisation**					
	Oitem11	All major strategic decisions need to be approved by top management.	0.89	0.83		
	Oitem12	We have to ask senior management before doing almost any decision.	0.62			
	Oitem14	Even quite small matters have to be referred to someone higher up for a final answer.	0.65			
	**Variable (V9): Formalisation**					
	Oitem15	We have a lot of rules and procedures stating how our job is to be done.	0.88	0.91		
	Oitem17	Whatever situation arises, we have procedures to follow.	0.87			
	Oitem18	The employees in your organisation are constantly checked for rule violation.	0.76			
	**Variable (V10): Perceived cost**					
	Oitem19	The implementation cost of HRIS is high for our organisation.	0.92	0.87		
	Oitem20	The direct and indirect cost for HRIS applications is high for our organisation.	0.77			
	Oitem21	The maintenance and support fees for HRIS applications are high for our company	0.86			
**Environment**	**Variable (V11): Competitive pressure**				0.00	83.14%
	Eitem1	Competitors’ adoption of HRIS places pressure on our organisation to adopt HRIS.	0.92	0.94		
	Eitem2	The overall operational practices in the industry pressure us to adopt HRIS.	0.91			
	Eitem3	Our organisation actively keeps track of new uses of technology by competitors.	0.94			
	**Variable (V12): Technology vendor support**					
	Eitem4	Training for HRIS is adequately provided by vendors.	0.87	0.85		
	Eitem5	Adequacy of technical support during HRIS implementation.	0.86			
	Eitem6	Adequacy of technical support after HRIS implementation.	0.92			
	**Variable (V13): Government regulations and support**					
	Eitem7	The availability of government security and protection influence us to use HRIS.	0.81	0.77		
	Eitem8	There are adequate financial aids from government (e.g. tax deduction, tariffs, financial subsidy) to adopt IT applications.	0.84			

### Results of the critical factors

The results of the present study indicate that all of the hypotheses are supported by ANOVA analysis. Particularly, the analysis results as shown in Table 4, strongly support hypotheses 2, 3,4,6,7 and 10 (p < 0.001), and also support hypotheses 5, 12 and 13 (p < 0.01). Additionally, the analysis results moderately support hypotheses 1 and 10 (p < 0.05).

**Table 4:** Results of statistical analysis.

Variables	F-Statistics	Adopter		Prospectors		Laggards	
		Mean	SD	Mean	SD	Mean	SD
Innovativeness of senior executives	6.01**	5.21	0.45	4.70	0.78	4.83	0.56
IT capabilities of staff	15.83***	5.70	0.12	5.92	0.34	6.31	0.57
IT Infrastructure	34.82***	5.93	0.34	6.33	0.23	6.57	0.56
Perceived compatibility	29.45***	6.11	0.73	5.79	0.87	5.30	0.45
Perceived complexity	4.09*	5.75	1.34	5.11	1.23	5.07	1.54
Relative advantage	17.34***	4.63	0.90	5.58	0.65	4.21	0.78
Top management support	27.48***	6.47	0.56	6.39	0.22	5.68	0.42
Centralisation	5.13*	6.22	0.89	5.37	1.10	5.30	0.78
Formalisation	6.78*	5.30	0.56	5.02	0.35	4.95	0.89
Perceived cost	10.83***	5.61	0.69	5.93	0.83	6.26	0.96
Competitive pressure	9.01**	6.41	0.34	5.40	0.12	5.81	0.45
Technology vendor support	3.78*	5.22	1.21	4.44	1.01	4.44	1.39
Government regulations and support	5.89*	4.88	0.78	4.31	0.67	4.34	0.29

*P<0.05

**p<0.01

***p<0.001

Also, the pair-wise mean differences of the 3 types of hospital pairs were analyzed and the analysis result is presented in Table 5. The results from this analysis represent that the adopters are significantly different from laggards in respect to all of the considered variables. However, the studied mean difference shows the diverse nature in cases of (prospectors, laggards), and (adopters, prospectors) pairs. Particularly, adopters are considerably different from prospectors in some factors i.e., innovativeness of senior executives, IT capabilities of staff, centralisation, formalisation, and technology vendor support. Prospectors are considerably different from laggards in relative advantage, perceived compatibility and also in perceived cost. The summary of results of pair-wise analysis is shown in Table 5.

**Table 5:** Results of pair-wise analysis.

Variables	Mean difference between adopters and laggard	Mean difference between adopters and prospectors	Mean difference between prospectors and laggards
Innovativeness of senior executives	Significant**	Significant*	Not Significant
IT capabilities of staff	Significant***	Significant***	Not Significant
IT Infrastructure	Significant***	Not Significant	Significant**
Perceived compatibility	Significant***	Not Significant	Significant***
Perceived complexity	Significant**	No Significant	Significant*
Relative advantage	Significant**	Not Significant	Significant*
Top management support	Significant***	Not Significant	Not Significant
Centralisation	Significant*	Not Significant	Significant*
Formalisation	Significant*	Not Significant	Not Significant
Perceived cost	Significant**	Not Significant	Significant*
Competitive pressure	Significant***	Not Significant	Not Significant
Technology vendor support	Significant**	Significant*	Not Significant
Government regulations and support	Significant*	Not Significant	Not Significant

*P<0.05

**p<0.01

***p<0.001

In this study, the 383 respondents indicate that the most critical factors are in descending order: consecutively IT infrastructure, top management support, IT capabilities of staff, perceived cost, competitive pressure, perceived compatibility, centralisation, perceived complexity, formalisation, innovativeness of senior executives, technology vendor support, relative advantage, and government regulations and support (Table 6). Among the 4 different dimensions, the technological dimension is recognized as the most important dimension (mean = 5.82), followed by organisational dimension (mean = 5.55), human dimension (mean = 5.50), and lastly environmental dimension (mean = 5.05).

**Table 6:** Overall analyses.

Dimensions	Mean	Rank	Variables	Mean	S.D.	Rank
Human	5.50	3	V1:Innovativeness of senior executives	4.98	0.65	10
			V2:IT capabilities of staff	6.01	0.47	3
Technological	5.82	1	V3:IT infrastructure	6.32	0.49	1
			V4: Perceived compatibility	5.76	0.74	6
			V5: Perceived complexity	5.36	0.45	8
Organisational	5.55	2	V6: Relative advantage	4.83	0.57	12
			V7:Top management support	6.21	0.76	2
			V8: Centralisation	5.67	0.82	7
			V9: Formalisation	5.12	0.69	9
			V10: Perceived cost	5.92	0.72	4
Environmental	5.05	4	V11:Competitive pressure	5.89	0.78	5
			V12:Technology vendor support	4.76	0.81	11
			V13:Government regulations and support	4.51	0.65	13

## Discussion

This research adds new knowledge to existing literature of HRIS adoption by investigative factors that influence its adoption decision in hospital industry of Bangladesh. The findings of the presented conceptual model induced diverse thought-provoking implications which are elucidated within the HOT fit model and in the TOE framework. Therefore, some assumptions can be drawn from the findings and the results of this study.

First, it is well accepted that personnel with IT knowledge and skills have a crucial role for adopting new IT applications in the organisations. So, the human dimension is an essential concern for any technology innovation adoption. It reflects the importance of innovativeness and capabilities of personnel in adopting a new technology. The present study includes 2 variables in this dimension, and they are innovativeness of senior executives and IT capabilities of staff. The first variable refers to the innovativeness—a personal characteristic—of the senior executives (CIO/CEO/owner) while the second one denotes the IS/IT competence of workforces. Achieved results represent the third most essential dimension among the suggested 4 dimensions (Table 5). Amongst the 13 factors, though innovativeness of senior executives is ranked at tenth position, it has a significant role for adopting new system in any hospital [22]. Earlier research is consistent with the present finding, and revealed that innovativeness and IT knowledge of CEOs reduces the degree of uncertainty for adopting new information systems [71]. Besides, IT capabilities of staff is identified as the third most significant factor among the 13 factors. Past studies also support its importance for HRIS adoption decision [13, 18, 19]. Similarly, Liu [21] stated that hospitals should cautiously evaluate their IS/IT capability of staff before finalizing the decision of adoption of technology. Since, a new information system changes the traditional working process of hospitals. It may require operating knowledge, and employees need to be aware of its impact. Sometimes, combining the new system with existing information systems is a required in a hospital. So, employee’s IT knowledge and skill will help to adjust themselves with the new platform of the hospital.

Second, the technological dimension is identified as the most important dimension for HRIS adoption in the hospitals. As expected, IT infrastructure and perceived compatibility are the two most dominant factors in the technology dimension. The present study reveals that IT infrastructure is the most significant (raked at first) factor to HRIS adoption in the hospitals of Bangladesh. This variable was also found significant in prior research on IT adoption in hospitals [49,55]. Similarly, Masum [14] also revealed IT infrastructure as a significant factor in Bangladesh setting. Other two factors—perceived compatibility and perceived complexity were ranked in sixth and eighth among the factors. In past studies, perceived compatibility was identified as a significant factor for HRIS adoption [1, 14], which is consistent with present studies. Sometimes, it is perceived wrongly that new IS may be incompatible with the present practice and culture of the organisation, or IS would create difficulties in the organisation [16]. So, hospital administration should have IT knowledge and experience, thus they can perceive right information about the system. However, recent research argued that perceived complexity was a less significant factor of HRIS adoption [1]. A probable cause would be that, nowadays, the employees are more computer literate and skilled with IT applications. Moreover, the perceived complexity of HRIS may be insignificant for the employees for their experience with other IS available in the organisations. In addition, IS and other softwares have become progressively easier to work with and are more user friendly.

Third, organisational dimension is revealed as the second most important dimension for adoption decision of HRIS in the hospitals of Bangladesh. This dimension contains 4 factors as per earlier discussion. Among them, top management support and perceived cost are identified as more important factors that influence hospital management decision for adopting HRIS. Results from this study show that top management support is the second most significant factor for HRIS adoption among the factors, which is consistent with aforementioned studies [1, 14, 70, 76, 77]. Prior scholars confirmed that HRIS has significant impact on work practices, and it changes the traditional HR activities. The researchers attested that top management support has critical role to overcome potential internal resistance after adopting HRIS. Also, top manager support ensures successful implementation of HRIS [1]. On the other hand, recent research stated that costs affect IT adoption decision and its use in the organisations [13,41]. With that same reasoning, the present study identified perceived cost as a fourth important factor for decision of HRIS adoption in hospitals. Al-Somali, Gholami et al. [70] argued that if the perceived costs (such as, cost for IT equipment, training cost and maintenance cost) are raised, it is more probable that overall IS practice will be less. However, obtained results show that the perceived benefits or relative advantages of HRIS adoption are comparatively insignificant to hospitals. This factor is widely recognized for adopting new technology innovation. But, the present outcome rejects the past research [1, 32, 40]. A possible reason would be that in this IT era, cost is the vital factor for any innovation in most of developing countries [14]. So, technological adoption may be delayed in spite of its immense advantages.

Fourth, the last dimension—environmental dimension—includes competitive pressure, technology vendor support, and government policy and support. This study represents that competitive pressure is identified as a fifth significant factor for HRIS adoption in hospitals of Bangladesh. The result is consistent with previous results. Past empirical evidence advocates that competitive pressure is an influential driver of IT adoption and diffusion [14, 45, 46]. However, the result shows disagreement on the study of Teo, Lim et al. [1], where competitive pressure was reported as insignificant factor. In addition, the environmental dimension and its factors result in relatively lower scores in compare to the other dimensions and their associated factors. According to above discussion, obtained results from this study shows that the most important dimension for HRIS adoption is technological dimension, followed by organisational, human, and finally environmental dimension. Furthermore, the most 5 critical concern for adopting HRIS is the IT infrastructure, top management support, IT capabilities of staff, perceived cost, and competitive pressure. Overall, the results are consistent with earlier research of IT innovation in other Asian nations [1, 13, 59, 78].

Fifth, the results show that there lies a significant difference between the adopters and the laggards in respect to all of the considered variables of HRIS adoption. The adopters also significantly differ from prospectors in innovativeness of senior executives, IT capabilities of staff, and technology vendor support. So, hospitals which are strongly supportive towards the uses of IT applications while developing IT assets (i.e., IT infrastructure, IT knowledge, IT capabilities) are the pioneer in adopting HRIS than the less IT supportive hospitals.

Sixth, the prospectors are significantly different from laggards technological dimension- IT infrastructure, compatibility, complexity; organisation dimension—relative advantages, centralisation, perceived cost—whereas there is no difference between adopters and prospectors in the technological and organisational dimensions. These results suggest that technological and organisational factors have an impact on attitudes toward HRIS, but do not have any effect on the relative earliness of adoption of HRIS. Therefore, the result shows its consistency with the model of “diffusion of innovation” by Rogers [27]. He stated that the perceived characteristic of intended innovation is the key factor on innovation decision process. The cost and benefit analysis is one of the reasons behind this phenomenon. Due to inadequate financial resources the Laggards remain in hesitation state regarding investment in adopting new technology such as HRIS. This is because, they are uncertain about the advantages of HRIS, the incompatibility of organisational existing ISs with modern HRIS, the culture, and the environment of the organisation. Then again, the adopters and the prospectors as well are eager in taking risks for HRIS adoption because of the recognized noticeable organizational performance due to HRIS adoption. Moreover, the certainty of compatible HRIS with the existing ISs of hospital is also their motivating factor of HRIS adoption. And, they are more certain on the compatibility of the HRIS with their hospitals.

Finally, the adopters’ nature of using HRIS in competitive environment differentiates the adopters from other 2 types of hospitals. Earlier research indicates that first movers take initiative to increase their competitive advantages more than laggards [13]. To gain the competitive advantages from HRIS adoption, adopters need adequate support from technology vendor and technology friendly government policies and support. This finding supports the past research that identified the influential environmental factors, which are essential for the management decision of successful adoption of any technology innovation such as HRIS [16, 28, 32, 47, 49, 59].

## Implications of the Study

HRIS adoption in developing countries is challenging because of the economical and infrastructural limitations. Moreover, social acceptance also hinders immediate HRIS adoption in the organisations. Therefore, a HRIS adoption model is proposed to explore the critical factors which stimulate the decision of adoption of HRIS in the hospitals of a developing country. The 5 influential critical factors are recognized as the most influential critical factors for adopting HRIS in hospitals of developing countries. And so, the research findings have multilateral implications while adopting HRIS in hospitals of developing countries. The implications of this research are discussed in following paragraphs.

HRIS enables numerous benefits to all of the stakeholders of hospitals. The impact of adopting the HRIS is explicit on the healthcare service delivery chain. Generally, HRIS helps the top management personnel in their strategic decision making process regarding recruitment, selection, placement, termination, training, development, and payroll. However, the HRIS for hospital management includes several subsystems for effective healthcare delivery [79]. The Hospital Information Subsystems (HIS) of HRIS provides information services to patients, guests, physicians, nurses and others by making necessary information available to the designated users. The modern Advanced Encryption Standard (AES) and access control policies enable the manageability along with the accessibility of information in HIS subsystems. Therefore, the HIS enables improved quality of experiences (qoe) to the end users which endures a social implication of HRIS. The Medical Expert Subsystem (MES) of HRIS aids the physicians in treatment planning and diseases diagnosis and the CADUCEUS and MYCIN are two popular MES of diseases diagnosis. Therefore, the MES offers practical implications in developing expertise to the physicians, consultants and surgeons. The Medical Case Management Subsystems (MCMS) of HRIS for hospital management enables the horizontal and vertical integration of healthcare providers and healthcare institutions or organizations. The case management system also facilitates the healthcare providers in treatment planning for the disable and injured individuals and also for the mental disordered individuals. Therefore, the MCMS endures the social implication especially for the minority subgroups, who needs special medical care. The Health Database Management Subsystem (HDMS) of Hospital Management Information System stores the structured and unstructured data of patients, treatments, diagnosis, medicines, and physicians as the patient history and trajectory. The researches and academicians use those data and process through Big-data mining approaches for finding interesting patterns of different diseases to improve the treatment systems. The Group Decision Support Subsystem (GDSS) of HRIS for hospital management supports the medical experts to work together in a virtual environment for analyzing the complex, critical and epidemic medical cases. Additionally, clinical decision support systems are also used for long-term patient care.

In conclusion, the HRIS for hospital management is clearly and explicitly adopting for an effective healthcare delivery. The drawn academic, practical and social implications of HRIS guide the top management authorities to build IT-infrastructures in hospitals, and motivate to recruit stuffs with sound IT capabilities. The HRIS also guides the management authorities to analyze the perceived cost of a modern Hospital Management Information System and also to analyze the competitive pressure from environment for gaining strategic business advantages.

## Conclusions

This research has extended the understanding of adoption behaviour by testing the phenomenon in a new environment. Hospital-level adoption behaviour of HRIS has never been investigated in Bangladesh and very little research has been conducted in similar developing countries. So, this research contributes to the existing body of knowledge by improving current understanding of HRIS adoption factors, which is an under-researched area in Bangladesh. This research mixes the eminent TOE framework with prominent HOT-fit technology innovation model to explore the most critical factors of influencing management decision in adopting the HRIS applications in hospitals. The discussion regarding the implications of HRIS for academia and practitioners are one of the foremost contributions of this research. In case of practitioners, this research reveals the significance of assumed factors that influence a hospital’s decision to adopt HRIS applications. Hospital managements can refer to the conclusions of this research to make a better decision on HRIS adoption. For academia, this research applies several earlier theories on technology adoption and diffusion to the field of HRIS, and found them consistent with obtained results. The main limitation of this research is the authors focus only on private hospitals of Bangladesh. There is no public/government hospitals data in the collected samples. So, the findings of this research have the limitation in terms of generalization. Moreover, this research is a cross-sectional study, and thus recommends a longitudinal study to explore the dynamics among the important factors and decision-making for HRIS adoption. This research only measured the major factors of human, technological, organisational, and environmental dimensions. Study on the samples from both private and public hospitals and inclusion of more factors will be the future extension towards the generalization of this study.

## Supporting Information

S1 File Data and variables of HRIS adoption survey. (PDF)
