# Supplementary material for: Critical Factors Influencing Decision to Adopt Human Resource Information System (HRIS) in Hospitals
Source: PLoS One. 2016 Aug 5;11(8):e0160366. doi: 10.1371/journal.pone.0160366 (PMC4975450; doi:10.1371/journal.pone.0160366)
Supplement: S1 File — (PDF) [file pone.0160366.s001.pdf]

S1 File. Data and variables of HRIS adoption survey

Demographic results

Respondent characteristic	Frequency	Percentage (%)
Gender		
Male	260	67.9
Female	123	32.1
Age		
26-31	29	7.6
31-39	124	32.3
39-45	147	38.4
45-58	83	21.7
Education level		
Bachelor’s	168	43.9
Master’s	189	49.3
M.Phil./PhD	26	6.8
Roles of respondents		
Senior Executive (HRM/IT)	145	37.9
Manager (HRM/IT)	238	62.1
Seniority in current position		
Above 15 years	16	4.1
10~14 years	47	12.4
5~9 years	173	45.1
1~4 years	124	32.4
Less than 1 year	23	6.0
Executives' seniority in the health care sector		
Above 26 years	35	9.1
21~25 years	57	14.9
16~20 years	50	13.0
11~15 years	74	19.3
6~10 years	75	19.6
Less than 5 years	92	24.1
HRIS adoption stage in hospitals		
Adopters	85	22.2
Prospectors	162	42.3
Laggards	136	35.5

Supporting Information

Response for job satisfaction factors

Response to questionnaire (1-8)

	Hitem1	Hitem2	Hitem3	Hitem4	Hitem5	Hitem6	Hitem7	Titem1	Titem2	Titem3	Titem4	Titem5	Titem6	Titem7	Titem8
Respondent 1	6	6	6	7	6	6	4	5	4	5	4	4	6	5	5
Respondent 2	5	6	5	6	5	6	6	5	5	6	6	6	5	5	5
Respondent 3	6	6	6	7	6	6	5	5	6	6	5	5	5	4	6
Respondent 4	4	5	4	5	4	7	5	5	4	5	5	5	6	5	4
Respondent 5	5	5	5	7	5	5	4	6	6	5	4	4	6	6	5
Respondent 6	5	6	5	6	5	6	5	4	5	5	5	5	5	6	5
Respondent 7	6	6	6	6	6	6	6	5	5	5	6	6	5	4	5
Respondent 8	4	5	4	7	4	7	6	5	4	6	6	6	5	6	6
Respondent 9	6	5	5	6	6	5	4	5	5	4	4	6	7	6	6
Respondent 10	5	5	6	6	5	7	6	6	6	5	6	5	6	5	6
Respondent 11	5	5	4	5	5	5	5	7	6	5	5	5	7	6	6
Respondent 12	4	6	6	5	4	6	5	5	4	5	5	6	5	4	7
Respondent 13	5	4	5	5	7	4	6	5	6	6	6	6	7	5	5
Respondent 14	6	5	5	5	6	7	5	4	5	7	5	5	6	5	6
Respondent 15	6	5	4	6	6	5	5	5	5	5	5	5	6	6	6
Respondent 16	4	5	5	7	7	5	5	6	6	5	5	5	7	4	7
Respondent 17	6	6	6	5	6	6	6	4	5	4	6	5	6	6	5
Respondent 18	5	7	6	5	5	7	3	4	5	5	3	6	6	5	7
Respondent 19	5	5	4	5	5	5	6	6	5	6	6	4	5	5	5
Respondent 20	6	5	6	6	7	5	5	6	6	4	5	5	5	4	6
Respondent 21	5	4	5	7	5	7	4	5	4	5	4	5	5	7	4
Respondent 22	5	5	5	5	5	5	6	5	5	6	6	5	5	6	7
Respondent 23	5	6	6	5	7	6	5	5	6	6	5	6	6	6	5
Respondent 24	6	4	5	7	6	7	5	5	4	5	5	7	7	7	5
Respondent 25	3	4	5	5	7	7	4	5	4	5	4	5	5	6	6
Respondent 26	6	6	5	6	6	6	6	5	5	6	6	5	5	5	7
Respondent 27	5	6	6	7	5	7	5	5	6	6	5	4	5	5	5
Respondent 28	6	6	3	7	6	6	5	5	4	5	5	5	7	6	6
Respondent 29	4	5	6	6	7	7	4	6	6	5	4	6	5	6	6
Respondent 30	3	3	1	4	5	5	5	4	5	5	5	4	5	5	7
Respondent 31	1	4	2	7	5	7	6	5	5	5	6	4	6	5	5
Respondent 32	2	2	3	3	6	6	6	5	4	6	6	3	4	6	5
Respondent 33	1	2	2	4	7	5	4	5	5	4	4	5	5	5	4

Respondent 34	7	6	6	4	2	7	6	6	4	3	3	5	7	6	6
Respondent 35	6	5	6	3	3	6	5	6	3	4	5	6	6	5	6
Respondent 36	7	6	6	4	4	7	6	6	3	2	5	3	7	6	6
Respondent 37	5	4	7	2	1	5	4	7	3	4	4	5	5	4	7
Respondent 38	7	5	5	2	2	7	5	5	4	2	3	5	7	5	5
Respondent 39	6	5	6	4	3	6	5	6	3	4	5	4	6	5	6
Respondent 40	6	6	6	1	2	6	6	6	5	3	6	3	6	6	6
Respondent 41	7	4	7	3	2	7	4	7	2	5	4	5	7	4	7
Respondent 42	6	6	5	4	1	6	6	5	3	4	3	3	6	6	5
Respondent 43	6	5	7	2	2	6	5	7	4	3	5	5	6	5	7
Respondent 44	5	5	5	2	2	5	5	5	2	3	2	5	5	5	5
Respondent 45	5	4	6	3	2	5	4	6	4	3	4	6	5	4	6
Respondent 46	5	7	4	4	3	5	7	4	2	4	3	3	5	7	4
Respondent 47	5	6	7	3	1	5	6	7	4	4	4	5	5	6	7
Respondent 48	6	6	5	4	2	6	6	5	7	6	6	7	6	6	5
Respondent 49	7	7	5	2	2	7	7	5	6	5	6	6	7	7	5
Respondent 50	5	6	6	2	2	5	6	6	7	6	6	7	5	6	6
Respondent 51	5	5	7	4	1	5	5	7	5	4	7	5	5	5	7
Respondent 52	5	5	5	1	2	5	5	5	7	5	5	7	5	5	5
Respondent 53	7	6	6	3	2	7	6	6	6	5	6	6	7	6	6
Respondent 54	6	5	6	4	2	6	5	6	6	6	6	6	6	5	6
Respondent 55	7	6	6	2	3	7	6	6	7	4	7	7	7	6	6
Respondent 56	5	4	7	2	4	5	4	7	6	6	5	6	5	4	7
Respondent 57	7	5	5	3	1	4	3	3	6	5	7	6	7	5	5
Respondent 58	4	3	3	4	2	3	4	4	5	5	5	5	6	5	6
Respondent 59	3	4	4	3	3	5	1	3	5	4	6	5	6	6	6
Respondent 60	4	2	2	4	2	1	3	4	5	7	4	5	7	4	7
Respondent 61	2	4	4	2	2	3	4	2	5	6	7	5	6	6	5
Respondent 62	2	2	2	2	1	4	2	2	6	6	5	6	6	5	7
Respondent 63	4	4	4	4	2	2	2	4	7	7	5	7	5	5	5
Respondent 64	1	3	3	1	2	3	4	1	5	6	6	5	5	4	6
Respondent 65	3	5	5	3	2	3	1	3	5	5	7	5	5	7	4
Respondent 66	4	2	4	4	3	4	3	4	5	5	5	5	5	6	7
Respondent 67	2	3	3	2	1	3	4	2	7	6	6	7	6	6	5
Respondent 68	2	3	3	2	2	5	2	2	6	5	6	6	7	7	5
Respondent 69	3	3	3	3	2	1	4	3	7	6	6	7	5	6	6
Respondent 70	4	4	4	4	2	3	3	4	5	4	7	5	5	5	7
Respondent 71	3	3	4	3	1	4	4	3	3	4	6	5	5	5	5
Respondent 72	4	3	3	4	2	2	2	4	3	3	4	6	7	6	6
Respondent 73	2	2	5	2	2	3	2	2	2	5	3	3	6	5	6
Respondent 74	4	3	2	4	2	1	4	4	3	2	5	5	7	6	6

Respondent 75	3	1	3	4	3	2	1	1	4	3	2	5	1	1	1
Respondent 76	5	6	5	6	5	6	6	5	5	6	6	6	5	5	5
Respondent 77	6	6	6	7	6	6	5	5	6	6	5	5	5	4	6
Respondent 78	4	5	4	5	4	7	5	5	4	5	5	5	6	5	4
Respondent 79	5	5	5	7	5	5	4	6	6	5	4	4	6	6	5
Respondent 80	5	6	5	6	5	6	5	4	5	5	5	5	5	6	5
Respondent 81	6	6	6	6	6	6	6	5	5	5	6	6	5	4	5
Respondent 82	4	5	4	7	4	7	6	5	4	6	6	6	5	6	6
Respondent 83	6	5	5	6	6	5	4	5	5	4	4	6	7	6	6
Respondent 84	5	5	6	6	5	7	6	6	6	5	6	5	6	5	6
Respondent 85	5	5	4	5	5	5	5	7	6	5	5	5	7	6	6
Respondent 86	4	6	6	5	4	6	5	5	4	5	5	6	5	4	7
Respondent 87	5	4	5	5	7	4	6	5	6	6	6	6	7	5	5
Respondent 88	6	5	5	5	6	7	5	4	5	7	5	5	6	5	6
Respondent 89	6	5	4	6	6	5	5	5	5	5	5	5	6	6	6
Respondent 90	4	5	5	7	7	5	5	6	6	5	5	5	7	4	7
Respondent 91	6	6	6	5	6	6	6	4	5	4	6	5	6	6	5
Respondent 92	5	7	6	5	5	7	3	4	5	5	3	6	6	5	7
Respondent 93	5	5	4	5	5	5	6	6	5	6	6	4	5	5	5
Respondent 94	6	5	6	6	7	5	5	6	6	4	5	5	5	4	6
Respondent 95	5	4	5	7	5	7	4	5	4	5	4	5	5	7	4
Respondent 96	5	5	5	5	5	5	6	5	5	6	6	5	5	6	7
Respondent 97	5	6	6	5	7	6	5	5	6	6	5	6	6	6	5
Respondent 98	6	4	5	7	6	7	5	5	4	5	5	7	7	7	5
Respondent 99	3	4	5	5	7	7	4	5	4	5	4	5	5	6	6
Respondent 100	6	6	5	6	6	6	6	5	5	6	6	5	5	5	7
Respondent 101	5	6	6	7	5	7	5	5	6	6	5	4	5	5	5
Respondent 102	6	6	3	7	6	6	5	5	4	5	5	5	7	6	6
Respondent 103	4	5	6	6	7	7	4	6	6	5	4	6	5	6	6
Respondent 104	3	3	1	4	5	5	5	4	5	5	5	4	5	5	7
Respondent 105	1	4	2	7	5	7	6	5	5	5	6	4	6	5	5
Respondent 106	2	2	3	3	6	6	6	5	4	6	6	3	4	6	5
Respondent 107	1	2	2	4	7	5	4	5	5	4	4	5	5	5	4
Respondent 108	5	6	5	6	5	6	6	5	5	6	6	6	5	5	5
Respondent 109	6	6	6	7	6	6	5	5	6	6	5	5	5	4	6
Respondent 110	4	5	4	5	4	7	5	5	4	5	5	5	6	5	4
Respondent 111	5	5	5	7	5	5	4	6	6	5	4	4	6	6	5
Respondent 112	5	6	5	6	5	6	5	4	5	5	5	5	5	6	5
Respondent 113	6	6	6	6	6	6	6	5	5	5	6	6	5	4	5
Respondent 114	4	5	4	7	4	7	6	5	4	6	6	6	5	6	6
Respondent 115	6	5	5	6	6	5	4	5	5	4	4	6	7	6	6

Respondent 116	7	6	6	4	2	3	2	3	3	3	3	3	6	6	5
Respondent 117	6	5	6	1	2	5	4	3	2	3	5	5	6	5	7
Respondent 118	7	6	6	3	2	1	3	4	3	3	3	5	5	5	5
Respondent 119	5	4	7	4	3	3	4	2	4	4	4	4	5	4	6
Respondent 120	7	5	5	1	3	4	2	2	2	3	2	5	5	7	4
Respondent 121	6	5	6	3	2	2	2	4	4	5	5	6	5	6	7
Respondent 122	6	6	6	4	2	3	4	1	2	2	5	5	6	6	5
Respondent 123	7	4	7	2	1	1	1	2	4	3	2	6	7	7	5
Respondent 124	6	6	5	2	2	2	3	4	3	4	3	3	5	6	6
Respondent 125	6	5	7	4	2	3	4	1	5	2	5	5	5	5	7
Respondent 126	5	5	5	1	2	4	2	3	4	4	4	5	5	5	5
Respondent 127	5	4	6	3	3	7	6	6	7	6	3	4	7	6	6
Respondent 128	5	7	4	4	3	6	5	6	6	5	4	3	5	6	6
Respondent 129	5	6	7	6	6	5	6	6	7	6	5	5	5	5	7
Respondent 130	6	6	5	6	5	7	4	7	5	4	4	5	6	5	5
Respondent 131	7	7	5	5	5	5	5	5	7	5	3	6	4	6	5
Respondent 132	5	6	6	5	4	6	5	6	6	5	5	5	5	5	4
Respondent 133	5	5	7	5	7	4	6	6	6	6	6	6	5	5	5
Respondent 134	5	5	5	5	6	7	4	7	7	4	4	3	7	6	6
Respondent 135	7	6	6	6	6	5	6	5	6	6	3	5	6	5	6
Respondent 136	6	5	6	7	7	5	5	7	6	5	5	5	7	6	6
Respondent 137	7	6	6	5	6	6	5	5	5	5	2	4	5	4	7
Respondent 138	5	4	7	5	5	7	4	6	5	4	4	3	7	5	5
Respondent 139	7	5	5	5	5	5	7	4	5	7	3	5	6	5	6
Respondent 140	6	5	6	7	6	6	6	7	5	6	5	5	6	6	6
Respondent 141	6	6	6	5	6	6	6	5	6	6	5	6	7	4	7
Respondent 142	7	4	7	5	5	7	7	5	7	7	4	5	6	6	5
Respondent 143	6	6	5	6	5	5	6	6	5	6	1	6	6	5	7
Respondent 144	6	5	7	4	6	5	5	7	5	5	2	3	5	5	5
Respondent 145	5	5	5	5	5	4	5	5	5	5	6	5	5	4	6
Respondent 146	1	3	4	5	5	5	6	6	7	6	4	5	5	7	4
Respondent 147	2	4	3	5	4	6	5	6	6	5	3	4	5	6	7
Respondent 148	2	1	1	6	5	4	6	6	7	6	5	5	6	6	5
Respondent 149	3	2	2	6	6	5	4	7	5	4	3	6	7	7	5
Respondent 150	3	3	3	4	2	7	5	5	7	5	4	4	5	6	6
Respondent 151	3	2	4	2	2	6	5	6	6	5	2	3	5	5	7
Respondent 152	1	2	1	4	3	6	6	6	6	6	5	5	5	5	5
Respondent 153	1	3	2	1	3	1	1	2	3	4	5	5	7	6	6
Respondent 154	1	3	2	3	2	3	3	2	5	3	2	6	6	5	6
Respondent 155	2	4	2	4	2	3	4	3	4	5	3	3	7	6	6
Respondent 156	3	3	3	2	1	4	2	4	3	2	5	5	5	4	7

Respondent 157	5	6	5	6	5	6	6	5	5	6	6	6	5	5	5
Respondent 158	6	6	6	7	6	6	5	5	6	6	5	5	5	4	6
Respondent 159	4	5	4	5	4	7	5	5	4	5	5	5	6	5	4
Respondent 160	5	5	5	7	5	5	4	6	6	5	4	4	6	6	5
Respondent 161	5	6	5	6	5	6	5	4	5	5	5	5	5	6	5
Respondent 162	6	6	6	6	6	6	6	5	5	5	6	6	5	4	5
Respondent 163	4	5	4	7	4	7	6	5	4	6	6	6	5	6	6
Respondent 164	6	5	5	6	6	5	4	5	5	4	4	6	7	6	6
Respondent 165	5	5	6	6	5	7	6	6	6	5	6	5	6	5	6
Respondent 166	5	5	4	5	5	5	5	7	6	5	5	5	7	6	6
Respondent 167	4	6	6	5	4	6	5	5	4	5	5	6	5	4	7
Respondent 168	5	4	5	5	7	4	6	5	6	6	6	6	7	5	5
Respondent 169	6	5	5	5	6	7	5	4	5	7	5	5	6	5	6
Respondent 170	6	5	4	6	6	5	5	5	5	5	5	5	6	6	6
Respondent 171	4	5	5	7	7	5	5	6	6	5	5	5	7	4	7
Respondent 172	6	6	6	5	6	6	6	4	5	4	6	5	6	6	5
Respondent 173	5	7	6	5	5	7	3	4	5	5	3	6	6	5	7
Respondent 174	5	5	4	5	5	5	6	6	5	6	6	4	5	5	5
Respondent 175	6	5	6	6	7	5	5	6	6	4	5	5	5	4	6
Respondent 176	5	4	5	7	5	7	4	5	4	5	4	5	5	7	4
Respondent 177	5	5	5	5	5	5	6	5	5	6	6	5	5	6	7
Respondent 178	5	6	6	5	7	6	5	5	6	6	5	6	6	6	5
Respondent 179	6	4	5	7	6	7	5	5	4	5	5	7	7	7	5
Respondent 180	3	4	5	5	7	7	4	5	4	5	4	5	5	6	6
Respondent 181	6	6	5	6	6	6	6	5	5	6	6	5	5	5	7
Respondent 182	5	6	6	7	5	7	5	5	6	6	5	4	5	5	5
Respondent 183	6	6	3	7	6	6	5	5	4	5	5	5	7	6	6
Respondent 184	4	5	6	6	7	7	4	6	6	5	4	6	5	6	6
Respondent 185	3	3	1	4	5	5	5	4	5	5	5	4	5	5	7
Respondent 186	1	4	2	7	5	7	6	5	5	5	6	4	6	5	5
Respondent 187	2	2	3	3	6	6	6	5	4	6	6	3	4	6	5
Respondent 188	1	2	2	4	7	5	4	5	5	4	4	5	5	5	4
Respondent 189	5	6	5	6	5	6	6	5	5	6	6	6	5	5	5
Respondent 190	6	6	6	7	6	6	5	5	6	6	5	5	5	4	6
Respondent 191	4	5	4	5	4	7	5	5	4	5	5	5	6	5	4
Respondent 192	5	5	5	7	5	5	4	6	6	5	4	4	6	6	5
Respondent 193	5	6	5	6	5	6	5	4	5	5	5	5	5	6	5
Respondent 194	6	6	6	6	6	6	6	5	5	5	6	6	5	4	5
Respondent 195	4	5	4	7	4	7	6	5	4	6	6	6	5	6	6
Respondent 196	6	5	5	6	6	5	4	5	5	4	4	6	7	6	6
Respondent 197	5	5	6	6	5	7	6	6	6	5	6	5	6	5	6

Respondent 198	2	4	3	3	3	4	4	3	3	4	3	6	6	6	5
Respondent 199	3	1	3	4	4	2	2	4	4	3	4	3	6	5	7
Respondent 200	3	2	4	2	1	3	2	2	2	5	2	5	5	5	5
Respondent 201	3	1	3	4	2	1	3	2	4	2	5	5	5	4	6
Respondent 202	5	6	5	6	5	6	6	5	5	6	6	6	5	5	5
Respondent 203	6	6	6	7	6	6	5	5	6	6	5	5	5	4	6
Respondent 204	4	5	4	5	4	7	5	5	4	5	5	5	6	5	4
Respondent 205	5	5	5	7	5	5	4	6	6	5	4	4	6	6	5
Respondent 206	5	6	5	6	5	6	5	4	5	5	5	5	5	6	5
Respondent 207	6	6	6	6	6	6	6	5	5	5	6	6	5	4	5
Respondent 208	4	5	4	7	4	7	6	5	4	6	6	6	5	6	6
Respondent 209	6	5	5	6	6	5	4	5	5	4	4	6	7	6	6
Respondent 210	5	5	6	6	5	7	6	6	6	5	6	5	6	5	6
Respondent 211	5	5	4	5	5	5	5	7	6	5	5	5	7	6	6
Respondent 212	4	6	6	5	4	6	5	5	4	5	5	6	5	4	7
Respondent 213	5	4	5	5	7	4	6	5	6	6	6	6	7	5	5
Respondent 214	6	5	5	5	6	7	5	4	5	7	5	5	6	5	6
Respondent 215	6	5	4	6	6	5	5	5	5	5	5	5	6	6	6
Respondent 216	4	5	5	7	7	5	5	6	6	5	5	5	7	4	7
Respondent 217	6	6	6	5	6	6	6	4	5	4	6	5	6	6	5
Respondent 218	5	7	6	5	5	7	3	4	5	5	3	6	6	5	7
Respondent 219	5	5	4	5	5	5	6	6	5	6	6	4	5	5	5
Respondent 220	6	5	6	6	7	5	5	6	6	4	5	5	5	4	6
Respondent 221	5	4	5	7	5	7	4	5	4	5	4	5	5	7	4
Respondent 222	5	5	5	5	5	5	6	5	5	6	6	5	5	6	7
Respondent 223	5	6	6	5	7	6	5	5	6	6	5	6	6	6	5
Respondent 224	6	4	5	7	6	7	5	5	4	5	5	7	7	7	5
Respondent 225	3	4	5	5	7	7	4	5	4	5	4	5	5	6	6
Respondent 226	6	6	5	6	6	6	6	5	5	6	6	5	5	5	7
Respondent 227	5	6	6	7	5	7	5	5	6	6	5	4	5	5	5
Respondent 228	6	6	3	7	6	6	5	5	4	5	5	5	7	6	6
Respondent 229	4	5	6	6	7	7	4	6	6	5	4	6	5	6	6
Respondent 230	3	3	1	4	5	5	5	4	5	5	5	4	5	5	7
Respondent 231	1	4	2	7	5	7	6	5	5	5	6	4	6	5	5
Respondent 232	2	2	3	3	6	6	6	5	4	6	6	3	4	6	5
Respondent 233	1	2	2	4	7	5	4	5	5	4	4	5	5	5	4
Respondent 234	5	6	5	6	5	6	6	5	5	6	6	6	5	5	5
Respondent 235	6	6	6	7	6	6	5	5	6	6	5	5	5	4	6
Respondent 236	4	5	4	5	4	7	5	5	4	5	5	5	6	5	4
Respondent 237	2	1	3	3	3	2	3	4	1	4	3	5	3	5	5
Respondent 238	1	2	4	4	1	3	4	2	3	3	4	5	4	6	5

Respondent 239	2	1	3	2	2	1	4	2	4	5	2	4	6	6	5
Respondent 240	3	2	1	2	2	2	1	3	1	4	4	3	6	5	7
Respondent 241	2	3	2	4	2	3	3	4	3	3	2	5	5	5	5
Respondent 242	2	4	3	1	3	4	4	1	3	3	3	5	5	4	6
Respondent 243	5	6	5	6	5	6	6	5	5	6	6	6	5	5	5
Respondent 244	6	6	6	7	6	6	5	5	6	6	5	5	5	4	6
Respondent 245	4	5	4	5	4	7	5	5	4	5	5	5	6	5	4
Respondent 246	5	5	5	7	5	5	4	6	6	5	4	4	6	6	5
Respondent 247	5	6	5	6	5	6	5	4	5	5	5	5	5	6	5
Respondent 248	6	6	6	6	6	6	6	5	5	5	6	6	5	4	5
Respondent 249	4	5	4	7	4	7	6	5	4	6	6	6	5	6	6
Respondent 250	6	5	5	6	6	5	4	5	5	4	4	6	7	6	6
Respondent 251	5	5	6	6	5	7	6	6	6	5	6	5	6	5	6
Respondent 252	5	5	4	5	5	5	5	7	6	5	5	5	7	6	6
Respondent 253	4	6	6	5	4	6	5	5	4	5	5	6	5	4	7
Respondent 254	5	4	5	5	7	4	6	5	6	6	6	6	7	5	5
Respondent 255	6	5	5	5	6	7	5	4	5	7	5	5	6	5	6
Respondent 256	6	5	4	6	6	5	5	5	5	5	5	5	6	6	6
Respondent 257	4	5	5	7	7	5	5	6	6	5	5	5	7	4	7
Respondent 258	6	6	6	5	6	6	6	4	5	4	6	5	6	6	5
Respondent 259	5	7	6	5	5	7	3	4	5	5	3	6	6	5	7
Respondent 260	5	5	4	5	5	5	6	6	5	6	6	4	5	5	5
Respondent 261	6	5	6	6	7	5	5	6	6	4	5	5	5	4	6
Respondent 262	5	4	5	7	5	7	4	5	4	5	4	5	5	7	4
Respondent 263	5	5	5	5	5	5	6	5	5	6	6	5	5	6	7
Respondent 264	5	6	6	5	7	6	5	5	6	6	5	6	6	6	5
Respondent 265	6	4	5	7	6	7	5	5	4	5	5	7	7	7	5
Respondent 266	3	4	5	5	7	7	4	5	4	5	4	5	5	6	6
Respondent 267	6	6	5	6	6	6	6	5	5	6	6	5	5	5	7
Respondent 268	5	6	6	7	5	7	5	5	6	6	5	4	5	5	5
Respondent 269	6	6	3	7	6	6	5	5	4	5	5	5	7	6	6
Respondent 270	4	5	6	6	7	7	4	6	6	5	4	6	5	6	6
Respondent 271	3	3	1	4	5	5	5	4	5	5	5	4	5	5	7
Respondent 272	1	4	2	7	5	7	6	5	5	5	6	4	6	5	5
Respondent 273	2	2	3	3	6	6	6	5	4	6	6	3	4	6	5
Respondent 274	1	2	2	4	7	5	4	5	5	4	4	5	5	5	4
Respondent 275	5	6	5	6	5	6	6	5	5	6	6	6	5	5	5
Respondent 276	6	6	6	7	6	6	5	5	6	6	5	5	5	4	6
Respondent 277	1	3	1	1	2	4	1	2	1	5	4	2	3	5	5
Respondent 278	1	2	2	3	2	2	3	2	3	2	3	3	4	6	5
Respondent 279	1	3	3	4	1	4	4	2	4	3	5	4	1	4	6

Respondent 280	2	4	4	2	2	3	2	3	2	4	2	1	3	5	5
Respondent 281	3	1	1	4	2	5	2	4	2	2	3	2	4	6	5
Respondent 282	4	2	2	2	2	1	3	3	4	4	4	3	1	4	6
Respondent 283	5	6	5	6	5	6	6	5	5	6	6	6	5	5	5
Respondent 284	6	6	6	7	6	6	5	5	6	6	5	5	5	4	6
Respondent 285	4	5	4	5	4	7	5	5	4	5	5	5	6	5	4
Respondent 286	5	5	5	7	5	5	4	6	6	5	4	4	6	6	5
Respondent 287	5	6	5	6	5	6	5	4	5	5	5	5	5	6	5
Respondent 288	6	6	6	6	6	6	6	5	5	5	6	6	5	4	5
Respondent 289	4	5	4	7	4	7	6	5	4	6	6	6	5	6	6
Respondent 290	6	5	5	6	6	5	4	5	5	4	4	6	7	6	6
Respondent 291	5	5	6	6	5	7	6	6	6	5	6	5	6	5	6
Respondent 292	5	5	4	5	5	5	5	7	6	5	5	5	7	6	6
Respondent 293	4	6	6	5	4	6	5	5	4	5	5	6	5	4	7
Respondent 294	5	4	5	5	7	4	6	5	6	6	6	6	7	5	5
Respondent 295	6	5	5	5	6	7	5	4	5	7	5	5	6	5	6
Respondent 296	6	5	4	6	6	5	5	5	5	5	5	5	6	6	6
Respondent 297	4	5	5	7	7	5	5	6	6	5	5	5	7	4	7
Respondent 298	6	6	6	5	6	6	6	4	5	4	6	5	6	6	5
Respondent 299	5	7	6	5	5	7	3	4	5	5	3	6	6	5	7
Respondent 300	5	5	4	5	5	5	6	6	5	6	6	4	5	5	5
Respondent 301	6	5	6	6	7	5	5	6	6	4	5	5	5	4	6
Respondent 302	5	4	5	7	5	7	4	5	4	5	4	5	5	7	4
Respondent 303	5	5	5	5	5	5	6	5	5	6	6	5	5	6	7
Respondent 304	5	6	6	5	7	6	5	5	6	6	5	6	6	6	5
Respondent 305	6	4	5	7	6	7	5	5	4	5	5	7	7	7	5
Respondent 306	3	4	5	5	7	7	4	5	4	5	4	5	5	6	6
Respondent 307	6	6	5	6	6	6	6	5	5	6	6	5	5	5	7
Respondent 308	5	6	6	7	5	7	5	5	6	6	5	4	5	5	5
Respondent 309	6	6	3	7	6	6	5	5	4	5	5	5	7	6	6
Respondent 310	4	5	6	6	7	7	4	6	6	5	4	6	5	6	6
Respondent 311	3	3	1	4	5	5	5	4	5	5	5	4	5	5	7
Respondent 312	1	4	2	7	5	7	6	5	5	5	6	4	6	5	5
Respondent 313	2	2	3	3	6	6	6	5	4	6	6	3	4	6	5
Respondent 314	1	2	2	4	7	5	4	5	5	4	4	5	5	5	4
Respondent 315	5	6	5	6	5	6	6	5	5	6	6	6	5	5	5
Respondent 316	6	6	6	7	6	6	5	5	6	6	5	5	5	4	6
Respondent 317	2	3	1	2	3	3	1	3	3	2	4	4	6	6	5
Respondent 318	3	4	2	2	2	4	3	4	4	3	2	3	6	5	7
Respondent 319	1	1	3	4	2	3	4	2	1	4	5	5	5	5	5
Respondent 320	2	2	4	1	3	5	4	2	3	2	5	4	5	4	6

Respondent 321	3	1	1	3	3	1	1	1	4	4	2	3	1	1	1
Respondent 322	4	3	3	4	3	3	3	3	2	2	3	5	3	3	3
Respondent 323	1	4	6	6	5	6	6	5	6	6	5	3	4	4	3
Respondent 324	3	2	6	5	7	6	5	7	6	5	4	4	2	2	4
Respondent 325	4	4	5	5	5	5	5	5	5	5	3	2	4	4	2
Respondent 326	2	3	5	4	6	5	4	6	5	4	5	5	2	3	2
Respondent 327	2	4	5	7	4	5	7	4	5	7	5	5	4	4	4
Respondent 328	4	2	5	6	7	5	6	7	5	6	4	2	3	2	1
Respondent 329	1	2	6	6	5	6	6	5	6	6	3	3	5	2	2
Respondent 330	3	4	7	7	5	7	7	5	7	7	5	2	1	4	4
Respondent 331	4	1	5	6	6	5	6	6	5	6	6	4	3	1	1
Respondent 332	4	3	5	5	7	5	5	7	5	5	4	3	4	3	3
Respondent 333	1	4	5	5	5	5	5	5	5	5	3	2	2	4	4
Respondent 334	3	2	7	6	6	7	6	6	7	6	5	5	3	2	2
Respondent 335	4	2	5	6	6	5	6	6	5	6	6	4	1	2	2
Respondent 336	2	3	5	5	7	5	5	7	5	5	4	3	2	3	3
Respondent 337	4	4	6	5	5	6	5	5	6	5	3	5	1	4	4
Respondent 338	3	1	4	6	5	4	6	5	4	6	5	6	3	1	1
Respondent 339	4	3	5	5	4	5	5	4	5	5	3	6	6	5	6
Respondent 340	2	4	5	5	5	5	5	5	5	5	4	6	5	7	6
Respondent 341	2	2	5	4	6	5	4	6	5	4	2	5	5	5	5
Respondent 342	3	2	6	5	4	6	5	4	6	5	5	5	4	6	5
Respondent 343	4	4	6	6	5	6	6	5	6	6	5	5	7	4	5
Respondent 344	1	1	6	5	7	6	5	7	6	5	2	5	6	7	5
Respondent 345	3	3	5	5	5	5	5	5	5	5	3	6	6	5	6
Respondent 346	4	4	5	4	6	5	4	6	5	4	5	7	7	5	7
Respondent 347	2	4	5	7	4	5	7	4	5	7	4	5	6	6	5
Respondent 348	2	1	5	6	7	5	6	7	5	6	3	5	5	7	5
Respondent 349	4	3	6	6	5	6	6	5	6	6	5	5	5	5	5
Respondent 350	1	4	7	7	5	7	7	5	7	7	5	7	6	6	7
Respondent 351	3	2	5	6	6	5	6	6	5	6	4	5	6	6	5
Respondent 352	4	4	5	5	7	5	5	7	5	5	3	5	5	7	5
Respondent 353	4	3	2	1	2	1	3	2	4	5	5	6	5	5	6
Respondent 354	1	4	3	3	1	3	4	3	1	4	3	4	6	5	4
Respondent 355	3	2	4	4	2	1	2	4	2	3	6	5	5	4	5
Respondent 356	4	2	1	2	2	3	2	1	4	3	3	5	5	5	5
Respondent 357	2	4	3	4	2	1	4	3	3	3	5	5	4	6	5
Respondent 358	4	1	4	1	3	2	1	4	4	4	4	6	5	4	6
Respondent 359	3	3	2	3	4	1	3	2	2	3	4	6	6	5	6
Respondent 360	4	4	2	4	1	3	4	2	2	5	3	6	5	7	6
Respondent 361	2	2	2	2	2	1	2	2	4	2	4	5	1	2	2

Respondent 362	5	6	5	6	5	6	6	5	5	6	6	6	5	5	5
Respondent 363	6	6	6	7	6	6	5	5	6	6	5	5	5	4	6
Respondent 364	4	5	4	5	4	7	5	5	4	5	5	5	6	5	4
Respondent 365	5	5	5	7	5	5	4	6	6	5	4	4	6	6	5
Respondent 366	5	6	5	6	5	6	5	4	5	5	5	5	5	6	5
Respondent 367	6	6	6	6	6	6	6	5	5	5	6	6	5	4	5
Respondent 368	4	5	4	7	4	7	6	5	4	6	6	6	5	6	6
Respondent 369	6	5	5	6	6	5	4	5	5	4	4	6	7	6	6
Respondent 370	5	5	6	6	5	7	6	6	6	5	6	5	6	5	6
Respondent 371	5	5	4	5	5	5	5	7	6	5	5	5	7	6	6
Respondent 372	4	6	6	5	4	6	5	5	4	5	5	6	5	4	7
Respondent 373	5	4	5	5	7	4	6	5	6	6	6	6	7	5	5
Respondent 374	6	5	5	5	6	7	5	4	5	7	5	5	6	5	6
Respondent 375	6	5	4	6	6	5	5	5	5	5	5	5	6	6	6
Respondent 376	4	5	5	7	7	5	5	6	6	5	5	5	7	4	7
Respondent 377	6	6	6	5	6	6	6	4	5	4	6	5	6	6	5
Respondent 378	5	7	6	5	5	7	3	4	5	5	3	6	6	5	7
Respondent 379	5	5	4	5	5	5	6	6	5	6	6	4	5	5	5
Respondent 380	6	5	6	6	7	5	5	6	6	4	5	5	5	4	6
Respondent 381	5	4	5	7	5	7	4	5	4	5	4	5	5	7	4
Respondent 382	5	5	5	5	5	5	6	5	5	6	6	5	5	6	7
Respondent 383	5	6	6	5	7	6	5	5	6	6	5	6	6	6	5

Response to questionnaire (9-23)

	Titem9	Tite10	Tite11	Tite12	Oitem1	Oitem1	Oitem2	Oitem3	Oitem4	Oitem5	Oitem6	Oitem7	Oitem8	Oitem9	Oitem10
Respondent 1	7	6	6	4	4	7	6	6	3	2	5	3	7	6	6
Respondent 2	5	4	7	2	1	5	4	7	3	4	4	5	5	4	7
Respondent 3	7	5	5	2	2	7	5	5	4	2	3	5	7	5	5
Respondent 4	7	6	6	4	4	7	6	6	3	2	5	3	7	6	6
Respondent 5	5	4	7	2	1	5	4	7	3	4	4	5	5	4	7
Respondent 6	5	6	5	6	5	6	5	4	5	5	5	5	5	6	5
Respondent 7	6	6	6	6	6	6	6	5	5	5	6	6	5	4	5
Respondent 8	4	5	4	7	4	7	6	5	4	6	6	6	5	6	6
Respondent 9	6	5	5	6	6	5	4	5	5	4	4	6	7	6	6
Respondent 10	5	5	6	6	5	7	6	6	6	5	6	5	6	5	6
Respondent 11	5	5	4	5	5	5	5	7	6	5	5	5	7	6	6
Respondent 12	4	6	6	5	4	6	5	5	4	5	5	6	5	4	7
Respondent 13	7	6	6	4	4	7	6	6	3	2	5	3	7	6	6
Respondent 14	5	4	7	2	1	5	4	7	3	4	4	5	5	4	7
Respondent 15	7	5	5	2	2	7	5	5	4	2	3	5	7	5	5
Respondent 16	7	6	6	4	4	7	6	6	3	2	5	3	7	6	6
Respondent 17	5	4	7	2	1	5	4	7	3	4	4	5	5	4	7
Respondent 18	7	5	5	2	2	7	5	5	4	2	3	5	7	5	5
Respondent 19	7	6	6	4	4	7	6	6	3	2	5	3	7	6	6
Respondent 20	5	4	7	2	1	5	4	7	3	4	4	5	5	4	7
Respondent 21	7	5	5	2	2	7	5	5	4	2	3	5	7	5	5
Respondent 22	5	5	5	5	5	5	6	5	5	6	6	5	5	6	7
Respondent 23	5	6	6	5	7	6	5	5	6	6	5	6	6	6	5
Respondent 24	6	4	5	7	6	7	5	5	4	5	5	7	7	7	5
Respondent 25	3	4	5	5	7	7	4	5	4	5	4	5	5	6	6
Respondent 26	6	6	5	6	6	6	6	5	5	6	6	5	5	5	7
Respondent 27	5	6	6	7	5	7	5	5	6	6	5	4	5	5	5
Respondent 28	6	6	3	7	6	6	5	5	4	5	5	5	7	6	6
Respondent 29	4	5	6	6	7	7	4	6	6	5	4	6	5	6	6
Respondent 30	3	3	1	4	5	5	5	4	5	5	5	4	5	5	7
Respondent 31	1	4	2	7	5	7	6	5	5	5	6	4	6	5	5
Respondent 32	2	2	3	3	6	6	6	5	4	6	6	3	4	6	5
Respondent 33	5	6	6	5	7	6	5	5	6	6	5	6	6	6	5
Respondent 34	6	4	5	7	6	7	5	5	4	5	5	7	7	7	5
Respondent 35	3	4	5	5	7	7	4	5	4	5	4	5	5	6	6
Respondent 36	6	6	5	6	6	6	6	5	5	6	6	5	5	5	7
Respondent 37	5	6	6	7	5	7	5	5	6	6	5	4	5	5	5
Respondent 38	6	6	3	7	6	6	5	5	4	5	5	5	7	6	6

Respondent 39	5	4	7	2	1	5	4	7	3	4	4	5	5	4	7
Respondent 40	7	5	5	2	2	7	5	5	4	2	3	5	7	5	5
Respondent 41	7	6	6	4	4	7	6	6	3	2	5	3	7	6	6
Respondent 42	5	4	7	2	1	5	4	7	3	4	4	5	5	4	7
Respondent 43	5	6	5	6	5	6	5	4	5	5	5	5	5	6	5
Respondent 44	6	6	6	6	6	6	6	5	5	5	6	6	5	4	5
Respondent 45	4	5	4	7	4	7	6	5	4	6	6	6	5	6	6
Respondent 46	6	5	5	6	6	5	4	5	5	4	4	6	7	6	6
Respondent 47	5	5	6	6	5	7	6	6	6	5	6	5	6	5	6
Respondent 48	5	5	4	5	5	5	5	7	6	5	5	5	7	6	6
Respondent 49	4	6	6	5	4	6	5	5	4	5	5	6	5	4	7
Respondent 50	7	6	6	4	4	7	6	6	3	2	5	3	7	6	6
Respondent 51	5	4	7	2	1	5	4	7	3	4	4	5	5	4	7
Respondent 52	7	5	5	2	2	7	5	5	4	2	3	5	7	5	5
Respondent 53	7	6	6	4	4	7	6	6	3	2	5	3	7	6	6
Respondent 54	5	4	7	2	1	5	4	7	3	4	4	5	5	4	7
Respondent 55	7	5	5	2	2	7	5	5	4	2	3	5	7	5	5
Respondent 56	7	6	6	4	4	7	6	6	3	2	5	3	7	6	6
Respondent 57	5	4	7	2	1	5	4	7	3	4	4	5	5	4	7
Respondent 58	7	5	5	2	2	7	5	5	4	2	3	5	7	5	5
Respondent 59	5	5	5	5	5	5	6	5	5	6	6	5	5	6	7
Respondent 60	5	6	6	5	7	6	5	5	6	6	5	6	6	6	5
Respondent 61	6	4	5	7	6	7	5	5	4	5	5	7	7	7	5
Respondent 62	3	4	5	5	7	7	4	5	4	5	4	5	5	6	6
Respondent 63	6	6	5	6	6	6	6	5	5	6	6	5	5	5	7
Respondent 64	5	6	6	7	5	7	5	5	6	6	5	4	5	5	5
Respondent 65	6	6	3	7	6	6	5	5	4	5	5	5	7	6	6
Respondent 66	4	5	6	6	7	7	4	6	6	5	4	6	5	6	6
Respondent 67	3	3	1	4	5	5	5	4	5	5	5	4	5	5	7
Respondent 68	1	4	2	7	5	7	6	5	5	5	6	4	6	5	5
Respondent 69	2	2	3	3	6	6	6	5	4	6	6	3	4	6	5
Respondent 70	5	6	6	5	7	6	5	5	6	6	5	6	6	6	5
Respondent 71	6	4	5	7	6	7	5	5	4	5	5	7	7	7	5
Respondent 72	3	4	5	5	7	7	4	5	4	5	4	5	5	6	6
Respondent 73	6	6	5	6	6	6	6	5	5	6	6	5	5	5	7
Respondent 74	5	6	6	7	5	7	5	5	6	6	5	4	5	5	5
Respondent 75	5	4	7	2	1	5	4	7	3	4	4	5	5	4	7
Respondent 76	7	5	5	2	2	7	5	5	4	2	3	5	7	5	5
Respondent 77	7	6	6	4	4	7	6	6	3	2	5	3	7	6	6
Respondent 78	5	4	7	2	1	5	4	7	3	4	4	5	5	4	7
Respondent 79	5	6	5	6	5	6	5	4	5	5	5	5	5	6	5

Respondent 80	5	6	5	6	5	6	5	4	5	5	5	5	5	6	5
Respondent 81	6	6	6	6	6	6	6	5	5	5	6	6	5	4	5
Respondent 82	4	5	4	7	4	7	6	5	4	6	6	6	5	6	6
Respondent 83	6	5	5	6	6	5	4	5	5	4	4	6	7	6	6
Respondent 84	5	5	6	6	5	7	6	6	6	5	6	5	6	5	6
Respondent 85	5	5	4	5	5	5	5	7	6	5	5	5	7	6	6
Respondent 86	5	4	7	2	1	5	4	7	3	4	4	5	5	4	7
Respondent 87	7	5	5	2	2	7	5	5	4	2	3	5	7	5	5
Respondent 88	7	6	6	4	4	7	6	6	3	2	5	3	7	6	6
Respondent 89	5	4	7	2	1	5	4	7	3	4	4	5	5	4	7
Respondent 90	5	6	5	6	5	6	5	4	5	5	5	5	5	6	5
Respondent 91	6	6	6	6	6	6	6	5	5	5	6	6	5	4	5
Respondent 92	4	5	4	7	4	7	6	5	4	6	6	6	5	6	6
Respondent 93	6	5	5	6	6	5	4	5	5	4	4	6	7	6	6
Respondent 94	5	5	6	6	5	7	6	6	6	5	6	5	6	5	6
Respondent 95	5	5	4	5	5	5	5	7	6	5	5	5	7	6	6
Respondent 96	4	6	6	5	4	6	5	5	4	5	5	6	5	4	7
Respondent 97	7	6	6	4	4	7	6	6	3	2	5	3	7	6	6
Respondent 98	5	4	7	2	1	5	4	7	3	4	4	5	5	4	7
Respondent 99	7	5	5	2	2	7	5	5	4	2	3	5	7	5	5
Respondent 100	7	6	6	4	4	7	6	6	3	2	5	3	7	6	6
Respondent 101	5	4	7	2	1	5	4	7	3	4	4	5	5	4	7
Respondent 102	7	5	5	2	2	7	5	5	4	2	3	5	7	5	5
Respondent 103	7	6	6	4	4	7	6	6	3	2	5	3	7	6	6
Respondent 104	5	4	7	2	1	5	4	7	3	4	4	5	5	4	7
Respondent 105	7	5	5	2	2	7	5	5	4	2	3	5	7	5	5
Respondent 106	5	5	5	5	5	5	6	5	5	6	6	5	5	6	7
Respondent 107	5	6	6	5	7	6	5	5	6	6	5	6	6	6	5
Respondent 108	6	4	5	7	6	7	5	5	4	5	5	7	7	7	5
Respondent 109	3	4	5	5	7	7	4	5	4	5	4	5	5	6	6
Respondent 110	6	6	5	6	6	6	6	5	5	6	6	5	5	5	7
Respondent 111	5	6	6	7	5	7	5	5	6	6	5	4	5	5	5
Respondent 112	6	6	3	7	6	6	5	5	4	5	5	5	7	6	6
Respondent 113	4	5	6	6	7	7	4	6	6	5	4	6	5	6	6
Respondent 114	3	3	1	4	5	5	5	4	5	5	5	4	5	5	7
Respondent 115	1	4	2	7	5	7	6	5	5	5	6	4	6	5	5
Respondent 116	2	2	3	3	6	6	6	5	4	6	6	3	4	6	5
Respondent 117	5	6	6	5	7	6	5	5	6	6	5	6	6	6	5
Respondent 118	7	6	6	3	2	1	3	4	3	3	3	5	5	5	5
Respondent 119	5	4	7	4	3	3	4	2	4	4	4	4	5	4	6
Respondent 120	7	5	5	1	3	4	2	2	2	3	2	5	5	7	4

Respondent 121	6	5	6	3	2	2	2	4	4	5	5	6	5	6	7
Respondent 122	6	6	6	4	2	3	4	1	2	2	5	5	6	6	5
Respondent 123	7	4	7	2	1	1	1	2	4	3	2	6	7	7	5
Respondent 124	6	6	5	2	2	2	3	4	3	4	3	3	5	6	6
Respondent 125	6	5	7	4	2	3	4	1	5	2	5	5	5	5	7
Respondent 126	5	5	5	1	2	4	2	3	4	4	4	5	5	5	5
Respondent 127	5	4	6	3	3	7	6	6	7	6	3	4	7	6	6
Respondent 128	5	7	4	4	3	6	5	6	6	5	4	3	5	6	6
Respondent 129	5	6	7	6	6	5	6	6	7	6	5	5	5	5	7
Respondent 130	6	6	5	6	5	7	4	7	5	4	4	5	6	5	5
Respondent 131	7	7	5	5	5	5	5	5	7	5	3	6	4	6	5
Respondent 132	5	6	6	5	4	6	5	6	6	5	5	5	5	5	4
Respondent 133	5	5	7	5	7	4	6	6	6	6	6	6	5	5	5
Respondent 134	5	5	5	5	6	7	4	7	7	4	4	3	7	6	6
Respondent 135	7	6	6	6	6	5	6	5	6	6	3	5	6	5	6
Respondent 136	6	5	6	7	7	5	5	7	6	5	5	5	7	6	6
Respondent 137	7	6	6	5	6	6	5	5	5	5	2	4	5	4	7
Respondent 138	5	4	7	5	5	7	4	6	5	4	4	3	7	5	5
Respondent 139	7	5	5	5	5	5	7	4	5	7	3	5	6	5	6
Respondent 140	6	5	6	7	6	6	6	7	5	6	5	5	6	6	6
Respondent 141	6	6	6	5	6	6	6	5	6	6	5	6	7	4	7
Respondent 142	7	4	7	5	5	7	7	5	7	7	4	5	6	6	5
Respondent 143	6	6	5	6	5	5	6	6	5	6	1	6	6	5	7
Respondent 144	6	5	7	4	6	5	5	7	5	5	2	3	5	5	5
Respondent 145	5	4	3	5	5	4	5	5	5	5	6	5	5	4	6
Respondent 146	6	5	5	5	6	5	6	6	7	6	4	5	5	7	4
Respondent 147	4	4	5	6	4	4	5	6	6	5	3	4	5	6	7
Respondent 148	5	3	6	4	5	3	6	6	7	6	5	5	6	6	5
Respondent 149	5	5	5	5	5	5	4	7	5	4	3	6	7	7	5
Respondent 150	6	6	6	5	6	6	5	5	7	5	4	4	5	6	6
Respondent 151	4	4	3	7	4	4	5	6	6	5	2	3	5	5	7
Respondent 152	6	3	5	6	6	3	6	6	6	6	5	5	5	5	5
Respondent 153	5	5	5	7	5	5	1	2	3	4	5	5	7	6	6
Respondent 154	5	2	4	5	5	2	3	2	5	3	2	6	6	5	6
Respondent 155	4	4	3	7	4	4	4	3	4	5	3	3	7	6	6
Respondent 156	7	3	5	6	7	3	2	4	3	2	5	5	5	4	7
Respondent 157	6	5	5	6	6	5	6	5	5	6	6	6	5	5	5
Respondent 158	6	5	6	7	6	5	5	5	6	6	5	5	5	4	6
Respondent 159	7	4	5	6	7	4	5	5	4	5	5	5	6	5	4
Respondent 160	6	1	6	6	6	1	4	6	6	5	4	4	6	6	5
Respondent 161	5	2	3	5	5	2	5	4	5	5	5	5	5	6	5

Respondent 162	5	4	3	5	5	4	3	5	5	4	3	5	5	4	3
Respondent 163	6	5	5	5	6	5	5	5	6	5	5	5	6	5	5
Respondent 164	4	4	5	6	4	4	5	6	4	4	5	6	4	4	5
Respondent 165	5	5	6	6	5	7	6	6	6	5	6	5	6	5	6
Respondent 166	5	5	4	5	5	5	5	7	6	5	5	5	7	6	6
Respondent 167	4	6	6	5	4	6	5	5	4	5	5	6	5	4	7
Respondent 168	5	4	5	5	7	4	6	5	6	6	6	6	7	5	5
Respondent 169	6	5	5	5	6	7	5	4	5	7	5	5	6	5	6
Respondent 170	6	5	4	6	6	5	5	5	5	5	5	5	6	6	6
Respondent 171	4	5	5	7	7	5	5	6	6	5	5	5	7	4	7
Respondent 172	6	6	6	5	6	6	6	4	5	4	6	5	6	6	5
Respondent 173	5	7	6	5	5	7	3	4	5	5	3	6	6	5	7
Respondent 174	5	5	4	5	5	5	6	6	5	6	6	4	5	5	5
Respondent 175	6	5	6	6	7	5	5	6	6	4	5	5	5	4	6
Respondent 176	5	4	5	7	5	7	4	5	4	5	4	5	5	7	4
Respondent 177	5	5	5	5	5	5	6	5	5	6	6	5	5	6	7
Respondent 178	5	6	6	5	7	6	5	5	6	6	5	6	6	6	5
Respondent 179	6	4	5	7	6	7	5	5	4	5	5	7	7	7	5
Respondent 180	3	4	5	5	7	7	4	5	4	5	4	5	5	6	6
Respondent 181	6	6	5	6	6	6	6	5	5	6	6	5	5	5	7
Respondent 182	5	6	6	7	5	7	5	5	6	6	5	4	5	5	5
Respondent 183	6	6	3	7	6	6	5	5	4	5	5	5	7	6	6
Respondent 184	4	5	6	6	7	7	4	6	6	5	4	6	5	6	6
Respondent 185	3	3	1	4	5	5	5	4	5	5	5	4	5	5	7
Respondent 186	1	4	2	7	5	7	6	5	5	5	6	4	6	5	5
Respondent 187	2	2	3	3	6	6	6	5	4	6	6	3	4	6	5
Respondent 188	1	2	2	4	7	5	4	5	5	4	4	5	5	5	4
Respondent 189	5	6	5	6	5	6	6	5	5	6	6	6	5	5	5
Respondent 190	6	6	6	7	6	6	5	5	6	6	5	5	5	4	6
Respondent 191	4	5	4	5	4	7	5	5	4	5	5	5	6	5	4
Respondent 192	5	5	5	7	5	5	4	6	6	5	4	4	6	6	5
Respondent 193	5	6	5	6	5	6	5	4	5	5	5	5	5	6	5
Respondent 194	6	6	6	6	6	6	6	5	5	5	6	6	5	4	5
Respondent 195	4	5	4	7	4	7	6	5	4	6	6	6	5	6	6
Respondent 196	6	5	5	6	6	5	4	5	5	4	4	6	7	6	6
Respondent 197	5	4	3	5	5	4	3	5	5	4	3	5	5	4	3
Respondent 198	6	5	5	5	6	5	5	5	6	5	5	5	6	5	5
Respondent 199	4	4	5	6	4	4	5	6	4	4	5	6	4	4	5
Respondent 200	5	3	6	4	5	3	6	4	5	3	6	4	5	3	6
Respondent 201	5	5	5	5	5	5	5	5	5	5	5	5	5	5	5
Respondent 202	6	6	6	5	6	6	6	5	6	6	6	5	6	6	6

Respondent 203	7	6	6	6	5	6	5	6	7	6	6	6	5	6	5
Respondent 204	5	5	7	6	5	5	5	7	5	5	7	6	5	5	5
Respondent 205	6	5	5	4	5	5	6	5	6	5	5	4	5	5	6
Respondent 206	4	6	5	6	6	6	6	7	4	6	5	6	6	6	6
Respondent 207	7	6	6	6	5	5	6	6	7	6	6	6	5	6	5
Respondent 208	4	5	4	7	4	6	6	3	7	6	6	6	5	6	6
Respondent 209	6	5	5	6	6	4	5	6	6	4	4	6	7	6	6
Respondent 210	5	6	6	7	5	3	3	1	4	6	5	4	5	5	5
Respondent 211	6	6	3	7	6	1	4	2	7	5	5	5	7	6	6
Respondent 212	4	5	6	6	7	5	6	6	7	5	4	6	5	6	6
Respondent 213	3	3	1	4	5	6	6	3	7	5	5	4	5	5	7
Respondent 214	1	4	2	7	5	4	5	6	6	5	6	4	6	5	5
Respondent 215	5	6	6	7	5	3	3	1	4	6	5	4	5	5	5
Respondent 216	6	6	3	7	6	1	4	2	7	5	5	5	7	6	6
Respondent 217	4	5	6	6	7	5	6	6	7	5	4	6	5	6	6
Respondent 218	7	6	6	6	5	6	6	3	7	6	6	6	5	6	5
Respondent 219	5	5	7	6	5	4	5	6	6	5	7	6	5	5	5
Respondent 220	6	5	5	4	5	3	3	1	4	5	5	4	5	5	6
Respondent 221	4	6	5	6	6	1	4	2	7	6	5	6	6	6	6
Respondent 222	7	6	6	6	5	5	6	6	7	6	6	6	5	6	5
Respondent 223	5	5	7	6	5	6	6	3	7	5	7	6	5	5	5
Respondent 224	6	5	5	4	5	4	5	6	6	5	5	4	5	5	6
Respondent 225	4	6	5	6	6	3	3	1	4	6	5	6	6	6	6
Respondent 226	6	6	5	6	6	1	4	2	7	6	6	5	5	5	7
Respondent 227	5	6	6	7	5	5	6	6	7	6	5	4	5	5	5
Respondent 228	6	6	3	7	6	6	6	3	7	5	5	5	7	6	6
Respondent 229	4	5	6	6	7	4	5	6	6	5	4	6	5	6	6
Respondent 230	3	3	1	4	5	3	3	1	4	5	5	4	5	5	7
Respondent 231	1	4	2	7	5	1	4	2	7	5	6	4	6	5	5
Respondent 232	2	2	3	3	6	5	6	6	7	6	6	3	4	6	5
Respondent 233	1	2	2	4	7	6	6	3	7	4	4	5	5	5	4
Respondent 234	5	6	5	6	5	4	5	6	6	6	6	6	5	5	5
Respondent 235	6	6	6	7	6	3	3	1	4	6	5	5	5	4	6
Respondent 236	4	5	4	5	4	1	4	2	7	5	5	5	6	5	4
Respondent 237	2	1	3	3	3	5	6	6	7	4	3	5	3	5	5
Respondent 238	1	2	4	4	1	6	6	3	7	3	4	5	4	6	5
Respondent 239	7	6	6	6	5	4	5	6	6	6	6	6	5	6	5
Respondent 240	5	5	7	6	5	3	3	1	4	5	7	6	5	5	5
Respondent 241	6	5	5	4	5	1	4	2	7	5	5	4	5	5	6
Respondent 242	4	6	5	6	6	5	6	6	7	6	5	6	6	6	6
Respondent 243	7	6	6	6	5	6	6	3	7	6	6	6	5	6	5

Respondent 244	6	5	6	6	7	5	5	6	6	4	5	5	5	4	6
Respondent 245	5	4	5	7	5	7	4	5	4	5	4	5	5	7	4
Respondent 246	5	5	5	5	5	5	6	5	5	6	6	5	5	6	7
Respondent 247	5	6	6	5	7	6	5	5	6	6	5	6	6	6	5
Respondent 248	6	4	5	7	6	7	5	5	4	5	5	7	7	7	5
Respondent 249	3	4	5	5	7	7	4	5	4	5	4	5	5	6	6
Respondent 250	6	5	5	6	6	5	4	5	5	4	4	6	7	6	6
Respondent 251	5	5	6	6	5	7	6	6	6	5	6	5	6	5	6
Respondent 252	5	5	4	5	5	5	5	7	6	5	5	5	7	6	6
Respondent 253	4	6	6	5	4	6	5	5	4	5	5	6	5	4	7
Respondent 254	5	4	5	5	7	4	6	5	6	6	6	6	7	5	5
Respondent 255	6	5	5	5	6	7	5	4	5	7	5	5	6	5	6
Respondent 256	6	5	4	6	6	5	5	5	5	5	5	5	6	6	6
Respondent 257	4	5	5	7	7	5	5	6	6	5	5	5	7	4	7
Respondent 258	6	6	6	5	6	6	6	4	5	4	6	5	6	6	5
Respondent 259	5	7	6	5	5	7	3	4	5	5	3	6	6	5	7
Respondent 260	5	5	4	5	5	5	6	6	5	6	6	4	5	5	5
Respondent 261	6	5	6	6	7	5	5	6	6	4	5	5	5	4	6
Respondent 262	5	4	5	7	5	7	4	5	4	5	4	5	5	7	4
Respondent 263	5	5	5	5	5	5	6	5	5	6	6	5	5	6	7
Respondent 264	5	6	6	5	7	6	5	5	6	6	5	6	6	6	5
Respondent 265	6	4	5	7	6	7	5	5	4	5	5	7	7	7	5
Respondent 266	3	4	5	5	7	7	4	5	4	5	4	5	5	6	6
Respondent 267	6	6	5	6	6	6	6	5	5	6	6	5	5	5	7
Respondent 268	5	6	6	7	5	7	5	5	6	6	5	4	5	5	5
Respondent 269	6	6	3	7	6	6	5	5	4	5	5	5	7	6	6
Respondent 270	4	5	6	6	7	7	4	6	6	5	4	6	5	6	6
Respondent 271	5	4	5	5	5	5	5	4	5	5	5	4	5	5	7
Respondent 272	7	6	6	7	5	7	6	5	5	5	6	4	6	5	5
Respondent 273	5	5	7	5	6	6	6	5	4	6	6	3	4	6	5
Respondent 274	6	5	5	6	7	5	4	5	5	4	4	5	5	5	4
Respondent 275	4	6	5	4	5	6	6	5	5	6	6	6	5	5	5
Respondent 276	7	5	4	7	6	6	5	5	6	6	5	5	5	4	6
Respondent 277	5	5	5	5	2	4	1	2	1	5	4	2	3	5	5
Respondent 278	5	5	6	5	2	2	3	2	3	2	3	3	4	6	5
Respondent 279	6	6	4	6	1	4	4	2	4	3	5	4	1	4	6
Respondent 280	7	3	4	7	7	5	5	6	6	4	5	5	5	4	6
Respondent 281	5	6	6	5	5	7	4	5	4	5	4	5	5	7	4
Respondent 282	5	5	6	5	5	5	6	5	5	6	6	5	5	6	7
Respondent 283	7	4	5	7	7	6	5	5	6	6	5	6	6	6	5
Respondent 284	5	6	5	5	6	7	5	5	4	5	5	7	7	7	5

Respondent 285	5	4	5	5	4	5	5	4	5	5	4	5	5	4	5
Respondent 286	7	6	6	7	6	6	7	6	6	7	6	6	7	6	6
Respondent 287	5	5	7	5	5	7	5	5	7	5	5	7	5	5	7
Respondent 288	6	5	5	6	5	5	6	5	5	6	5	5	6	5	5
Respondent 289	4	6	5	4	6	5	4	6	5	4	6	5	4	6	5
Respondent 290	7	5	4	7	5	4	7	5	4	7	5	4	7	5	4
Respondent 291	5	5	6	6	5	7	6	6	6	5	6	5	6	5	6
Respondent 292	5	5	4	5	5	5	5	7	6	5	5	5	7	6	6
Respondent 293	4	6	6	5	4	6	5	5	4	5	5	6	5	4	7
Respondent 294	5	4	5	5	7	4	6	5	6	6	6	6	7	5	5
Respondent 295	6	5	5	5	6	7	5	4	5	7	5	5	6	5	6
Respondent 296	6	5	4	6	6	5	5	5	5	5	5	5	6	6	6
Respondent 297	4	5	5	7	7	5	5	6	6	5	5	5	7	4	7
Respondent 298	6	6	6	5	6	6	6	4	5	4	6	5	6	6	5
Respondent 299	5	7	6	5	5	7	3	4	5	5	3	6	6	5	7
Respondent 300	5	5	4	5	5	5	6	6	5	6	6	4	5	5	5
Respondent 301	6	5	6	6	7	5	5	6	6	4	5	5	5	4	6
Respondent 302	5	4	5	7	5	7	4	5	4	5	4	5	5	7	4
Respondent 303	5	5	5	5	5	5	6	5	5	6	6	5	5	6	7
Respondent 304	5	6	6	5	7	6	5	5	6	6	5	6	6	6	5
Respondent 305	6	4	5	7	6	7	5	5	4	5	5	7	7	7	5
Respondent 306	3	4	5	5	7	7	4	5	4	5	4	5	5	6	6
Respondent 307	6	6	5	6	6	6	6	5	5	6	6	5	5	5	7
Respondent 308	5	6	6	7	5	7	5	5	6	6	5	4	5	5	5
Respondent 309	6	6	3	7	6	6	5	5	4	5	5	5	7	6	6
Respondent 310	4	5	6	6	5	4	5	5	4	5	4	6	5	6	6
Respondent 311	3	3	1	4	7	6	6	7	6	6	5	4	5	5	7
Respondent 312	1	4	2	7	5	5	7	5	5	7	6	4	6	5	5
Respondent 313	2	2	3	3	6	5	5	6	5	5	6	3	4	6	5
Respondent 314	1	2	2	4	4	6	5	4	6	5	4	5	5	5	4
Respondent 315	5	6	5	6	7	5	4	7	5	4	6	6	5	5	5
Respondent 316	6	6	6	7	5	5	5	5	5	5	5	5	5	4	6
Respondent 317	2	3	1	2	5	5	6	5	5	6	4	4	6	6	5
Respondent 318	3	4	2	2	6	6	4	6	6	4	2	3	6	5	7
Respondent 319	1	1	3	4	7	3	4	7	3	4	5	5	5	5	5
Respondent 320	2	2	4	1	5	6	6	5	6	6	5	4	5	4	6
Respondent 321	3	1	1	3	5	5	6	5	5	6	2	3	1	1	1
Respondent 322	5	4	5	5	4	5	5	4	5	5	4	5	5	4	5
Respondent 323	7	6	6	7	6	6	7	6	6	7	6	6	7	6	6
Respondent 324	5	5	7	5	5	7	5	5	7	5	5	7	5	5	7
Respondent 325	6	5	5	6	5	5	6	5	5	6	5	5	6	5	5

Respondent 326	5	4	5	5	4	5	5	4	5	5	4	5	5	4	5
Respondent 327	7	6	6	7	6	6	7	6	6	7	6	6	7	6	6
Respondent 328	5	5	7	5	5	7	5	5	7	5	5	7	5	5	7
Respondent 329	6	5	5	6	5	5	6	5	5	6	5	5	6	5	5
Respondent 330	3	4	7	7	5	7	7	5	7	7	5	2	1	4	4
Respondent 331	4	1	5	6	6	5	6	6	5	6	6	4	3	1	1
Respondent 332	4	3	5	5	7	5	5	7	5	5	4	3	4	3	3
Respondent 333	1	4	5	5	5	5	5	5	5	5	3	2	2	4	4
Respondent 334	3	2	7	6	6	7	6	6	7	6	5	5	3	2	2
Respondent 335	4	2	5	6	6	5	6	6	5	6	6	4	1	2	2
Respondent 336	2	3	5	5	7	5	5	7	5	5	4	3	2	3	3
Respondent 337	4	4	6	5	5	6	5	5	6	5	3	5	1	4	4
Respondent 338	3	1	4	6	5	4	6	5	4	6	5	6	3	1	1
Respondent 339	4	3	5	5	4	5	5	4	5	5	3	6	6	5	6
Respondent 340	6	5	5	5	6	7	5	5	5	5	4	6	5	7	6
Respondent 341	6	5	4	6	6	5	5	6	5	4	2	5	5	5	5
Respondent 342	4	5	5	7	7	5	5	4	6	5	5	5	4	6	5
Respondent 343	6	6	6	5	6	6	6	5	6	6	5	5	7	4	5
Respondent 344	6	5	5	5	6	7	5	7	6	5	2	5	6	7	5
Respondent 345	6	5	4	6	6	5	5	5	5	5	3	6	6	5	6
Respondent 346	4	5	5	7	7	5	5	6	5	4	5	7	7	5	7
Respondent 347	6	6	6	5	6	6	6	4	5	7	4	5	6	6	5
Respondent 348	6	5	5	5	6	7	5	7	5	6	3	5	5	7	5
Respondent 349	6	5	4	6	6	5	5	5	6	6	5	5	5	5	5
Respondent 350	4	5	5	7	7	5	5	5	7	7	5	7	6	6	7
Respondent 351	6	6	6	5	6	6	6	6	5	6	4	5	6	6	5
Respondent 352	6	5	5	5	6	7	5	7	5	5	3	5	5	7	5
Respondent 353	6	5	4	6	6	5	5	2	4	5	5	6	5	5	6
Respondent 354	4	5	5	7	7	5	5	3	1	4	3	4	6	5	4
Respondent 355	6	6	6	5	6	6	6	4	2	3	6	5	5	4	5
Respondent 356	6	5	5	5	6	7	5	1	4	3	3	5	5	5	5
Respondent 357	6	5	4	6	6	5	5	3	3	3	5	5	4	6	5
Respondent 358	4	5	5	7	7	5	5	4	4	4	4	6	5	4	6
Respondent 359	6	6	6	5	6	6	6	2	2	3	4	6	6	5	6
Respondent 360	6	5	5	5	6	7	5	2	2	5	3	6	5	7	6
Respondent 361	6	5	4	6	6	5	5	2	4	2	4	5	1	2	2
Respondent 362	4	5	5	7	7	5	5	3	5	5	7	5	6	3	5
Respondent 363	6	5	5	5	5	5	6	5	5	5	5	5	6	5	5
Respondent 364	7	5	7	6	6	7	7	5	7	6	6	7	7	5	7
Respondent 365	6	4	5	6	6	5	6	4	5	6	6	5	6	4	5
Respondent 366	5	3	5	5	7	5	5	3	5	5	7	5	5	3	5

Respondent 367	6	3	5	5	7	5	6	3	5	5	7	5	6	3	5
Respondent 368	6	5	5	5	5	5	6	5	5	5	5	5	6	5	5
Respondent 369	7	5	7	6	6	7	7	5	7	6	6	7	7	5	7
Respondent 370	6	4	5	6	6	5	6	4	5	6	6	5	6	4	5
Respondent 371	5	5	4	5	5	5	5	7	6	5	5	5	7	6	6
Respondent 372	4	6	6	5	4	6	5	5	4	5	5	6	5	4	7
Respondent 373	5	4	5	5	7	4	6	5	6	6	6	6	7	5	5
Respondent 374	6	5	5	5	6	7	5	4	5	7	5	5	6	5	6
Respondent 375	6	5	4	6	6	5	5	5	5	5	5	5	6	6	6
Respondent 376	4	5	5	7	7	5	5	6	6	5	5	5	7	4	7
Respondent 377	6	6	6	5	6	6	6	4	5	4	6	5	6	6	5
Respondent 378	5	7	6	5	5	7	3	4	5	5	3	6	6	5	7
Respondent 379	6	3	5	5	7	5	6	3	5	5	7	5	6	3	5
Respondent 380	6	5	5	5	5	5	6	5	5	5	5	5	6	5	5
Respondent 381	7	5	7	6	6	7	7	5	7	6	6	7	7	5	7
Respondent 382	6	4	5	6	6	5	6	4	5	6	6	5	6	4	5
Respondent 383	5	3	5	5	7	5	5	3	5	5	7	5	5	3	5

Response to questionnaire (25-35)

	Oitem11	Oitem12	Oitem13	Oitem14	Oitem15	Oitem16	Oitem17	Oitem18	Oitem19	Oitem20	Oitem21
Respondent 1	5	4	7	2	1	5	4	7	3	4	4
Respondent 2	7	5	5	2	2	7	5	5	4	2	3
Respondent 3	7	6	6	4	4	7	6	6	3	2	5
Respondent 4	5	4	7	2	1	5	4	7	3	4	4
Respondent 5	7	5	5	2	2	7	5	5	4	2	3
Respondent 6	5	6	5	6	5	6	5	4	5	5	5
Respondent 7	6	6	6	6	6	6	6	5	5	5	6
Respondent 8	4	5	4	7	4	7	6	5	4	6	6
Respondent 9	6	5	5	6	6	5	4	5	5	4	4
Respondent 10	5	5	6	6	5	7	6	6	6	5	6
Respondent 11	5	5	4	5	5	5	5	7	6	5	5
Respondent 12	4	6	6	5	4	6	5	5	4	5	5
Respondent 13	7	6	6	4	4	7	6	6	3	2	5
Respondent 14	5	4	7	2	1	5	4	7	3	4	4
Respondent 15	7	5	5	2	2	7	5	5	4	2	3
Respondent 16	7	6	6	4	4	7	6	6	3	2	5
Respondent 17	5	4	7	2	1	5	4	7	3	4	4
Respondent 18	7	5	5	2	2	7	5	5	4	2	3
Respondent 19	7	6	6	4	4	7	6	6	3	2	5
Respondent 20	5	4	7	2	1	5	4	7	3	4	4
Respondent 21	7	5	5	2	2	7	5	5	4	2	3
Respondent 22	5	5	5	5	5	5	6	5	5	6	6
Respondent 23	5	6	6	5	7	6	5	5	6	6	5
Respondent 24	6	4	5	7	6	7	5	5	4	5	5
Respondent 25	3	4	5	5	7	7	4	5	4	5	4
Respondent 26	6	6	5	6	6	6	6	5	5	6	6
Respondent 27	5	6	6	7	5	7	5	5	6	6	5
Respondent 28	6	6	3	7	6	6	5	5	4	5	5
Respondent 29	4	5	6	6	7	7	4	6	6	5	4
Respondent 30	3	3	1	4	5	5	5	4	5	5	5
Respondent 31	1	4	2	7	5	7	6	5	5	5	6
Respondent 32	2	2	3	3	6	6	6	5	4	6	6
Respondent 33	5	4	7	2	1	5	4	7	3	4	4
Respondent 34	7	5	5	2	2	7	5	5	4	2	3
Respondent 35	7	6	6	4	4	7	6	6	3	2	5
Respondent 36	5	4	7	2	1	5	4	7	3	4	4
Respondent 37	7	5	5	2	2	7	5	5	4	2	3
Respondent 38	5	5	5	5	5	5	6	5	5	6	6

Respondent 39	5	2	2	7	5	5	4	2	5	2	2
Respondent 40	6	4	4	7	6	6	3	2	6	4	4
Respondent 41	7	2	1	5	4	7	3	4	7	2	1
Respondent 42	5	2	2	7	5	5	4	2	5	2	2
Respondent 43	5	2	2	7	5	5	4	2	5	2	2
Respondent 44	6	4	4	7	6	6	3	2	6	4	4
Respondent 45	7	2	1	5	4	7	3	4	7	2	1
Respondent 46	5	2	2	7	5	5	4	2	5	2	2
Respondent 47	5	5	6	6	5	7	6	6	6	5	6
Respondent 48	5	5	4	5	5	5	5	7	6	5	5
Respondent 49	4	6	6	5	4	6	5	5	4	5	5
Respondent 50	7	6	6	4	4	7	6	6	3	2	5
Respondent 51	5	4	7	2	1	5	4	7	3	4	4
Respondent 52	7	5	5	2	2	7	5	5	4	2	3
Respondent 53	7	6	6	4	4	7	6	6	3	2	5
Respondent 54	5	4	7	2	1	5	4	7	3	4	4
Respondent 55	7	5	5	2	2	7	5	5	4	2	3
Respondent 56	7	6	6	4	4	7	6	6	3	2	5
Respondent 57	5	4	7	2	1	5	4	7	3	4	4
Respondent 58	7	5	5	2	2	7	5	5	4	2	3
Respondent 59	5	5	5	5	5	5	6	5	5	6	6
Respondent 60	5	6	6	5	7	6	5	5	6	6	5
Respondent 61	6	4	5	7	6	7	5	5	4	5	5
Respondent 62	3	4	5	5	7	7	4	5	4	5	4
Respondent 63	6	6	5	6	6	6	6	5	5	6	6
Respondent 64	5	6	6	7	5	7	5	5	6	6	5
Respondent 65	6	6	3	7	6	6	5	5	4	5	5
Respondent 66	4	5	6	6	7	7	4	6	6	5	4
Respondent 67	3	3	1	4	5	5	5	4	5	5	5
Respondent 68	1	4	2	7	5	7	6	5	5	5	6
Respondent 69	2	2	3	3	6	6	6	5	4	6	6
Respondent 70	5	6	6	5	7	6	5	5	6	6	5
Respondent 71	6	4	5	7	6	7	5	5	4	5	5
Respondent 72	3	4	5	5	7	7	4	5	4	5	4
Respondent 73	6	6	5	6	6	6	6	5	5	6	6
Respondent 74	5	6	6	7	5	7	5	5	6	6	5
Respondent 75	5	2	2	7	5	5	4	2	5	2	2
Respondent 76	6	4	4	7	6	6	3	2	6	4	4
Respondent 77	7	2	1	5	4	7	3	4	7	2	1
Respondent 78	5	2	2	7	5	5	4	2	5	2	2
Respondent 79	5	2	2	7	5	5	4	2	5	2	2

Respondent 80	5	2	2	7	5	5	4	2	5	2	2
Respondent 81	6	4	4	7	6	6	3	2	6	4	4
Respondent 82	7	2	1	5	4	7	3	4	7	2	1
Respondent 83	5	2	2	7	5	5	4	2	5	2	2
Respondent 84	5	2	2	7	5	5	4	2	5	2	2
Respondent 85	5	5	4	5	5	5	5	7	6	5	5
Respondent 86	5	4	7	2	1	5	4	7	3	4	4
Respondent 87	7	5	5	2	2	7	5	5	4	2	3
Respondent 88	7	6	6	4	4	7	6	6	3	2	5
Respondent 89	5	4	7	2	1	5	4	7	3	4	4
Respondent 90	5	6	5	6	5	6	5	4	5	5	5
Respondent 91	6	6	6	6	6	6	6	5	5	5	6
Respondent 92	4	5	4	7	4	7	6	5	4	6	6
Respondent 93	6	5	5	6	6	5	4	5	5	4	4
Respondent 94	5	5	6	6	5	7	6	6	6	5	6
Respondent 95	5	2	2	7	5	5	4	2	5	2	2
Respondent 96	6	4	4	7	6	6	3	2	6	4	4
Respondent 97	7	2	1	5	4	7	3	4	7	2	1
Respondent 98	5	2	2	7	5	5	4	2	5	2	2
Respondent 99	5	2	2	7	5	5	4	2	5	2	2
Respondent 100	7	6	6	4	4	7	6	6	3	2	5
Respondent 101	5	4	7	2	1	5	4	7	3	4	4
Respondent 102	7	5	5	2	2	7	5	5	4	2	3
Respondent 103	7	6	6	4	4	7	6	6	3	2	5
Respondent 104	5	4	7	2	1	5	4	7	3	4	4
Respondent 105	7	5	5	2	2	7	5	5	4	2	3
Respondent 106	5	5	5	5	5	5	6	5	5	6	6
Respondent 107	5	6	6	5	7	6	5	5	6	6	5
Respondent 108	6	4	5	7	6	7	5	5	4	5	5
Respondent 109	3	4	5	5	7	7	4	5	4	5	4
Respondent 110	6	6	5	6	6	6	6	5	5	6	6
Respondent 111	5	6	6	7	5	7	5	5	6	6	5
Respondent 112	6	6	3	7	6	6	5	5	4	5	5
Respondent 113	4	5	6	6	7	7	4	6	6	5	4
Respondent 114	3	3	1	4	5	5	5	4	5	5	5
Respondent 115	1	4	2	7	5	7	6	5	5	5	6
Respondent 116	5	2	2	7	5	5	4	2	5	2	2
Respondent 117	6	4	4	7	6	6	3	2	6	4	4
Respondent 118	7	2	1	5	4	7	3	4	7	2	1
Respondent 119	5	2	2	7	5	5	4	2	5	2	2
Respondent 120	5	2	2	7	5	5	4	2	5	2	2

Respondent 121	5	6	6	5	4	6	5	6	6	5	5
Respondent 122	5	5	7	5	7	4	6	6	6	6	6
Respondent 123	5	5	5	5	6	7	4	7	7	4	4
Respondent 124	7	6	6	6	6	5	6	5	6	6	3
Respondent 125	5	6	6	5	4	6	5	6	6	5	5
Respondent 126	5	5	5	1	2	4	2	3	4	4	4
Respondent 127	5	4	6	3	3	7	6	6	7	6	3
Respondent 128	5	7	4	4	3	6	5	6	6	5	4
Respondent 129	5	6	7	6	6	5	6	6	7	6	5
Respondent 130	6	6	5	6	5	7	4	7	5	4	4
Respondent 131	7	7	5	5	5	5	5	5	7	5	3
Respondent 132	5	6	6	5	4	6	5	6	6	5	5
Respondent 133	5	5	7	5	7	4	6	6	6	6	6
Respondent 134	5	5	5	5	6	7	4	7	7	4	4
Respondent 135	7	6	6	6	6	5	6	5	6	6	3
Respondent 136	6	5	6	7	7	5	5	7	6	5	5
Respondent 137	7	6	6	5	6	6	5	5	5	5	2
Respondent 138	5	4	7	5	5	7	4	6	5	4	4
Respondent 139	7	5	5	5	5	5	7	4	5	7	3
Respondent 140	6	5	6	7	6	6	6	7	5	6	5
Respondent 141	6	6	6	5	6	6	6	5	6	6	5
Respondent 142	7	4	7	5	5	7	7	5	7	7	4
Respondent 143	6	6	5	6	5	5	6	6	5	6	1
Respondent 144	6	5	7	4	6	5	5	7	5	5	2
Respondent 145	5	4	3	5	5	4	5	5	5	5	6
Respondent 146	6	5	5	5	6	5	6	6	7	6	4
Respondent 147	4	4	5	6	4	4	5	6	6	5	3
Respondent 148	5	3	6	4	5	3	6	6	7	6	5
Respondent 149	5	5	5	5	5	5	4	7	5	4	3
Respondent 150	6	6	6	5	6	6	5	5	7	5	4
Respondent 151	4	4	3	7	4	4	5	6	6	5	2
Respondent 152	6	3	5	6	6	3	6	6	6	6	5
Respondent 153	5	5	5	7	5	5	1	2	3	4	5
Respondent 154	5	2	4	5	5	2	3	2	5	3	2
Respondent 155	4	4	3	7	4	4	4	3	4	5	3
Respondent 156	7	3	5	6	7	3	2	4	3	2	5
Respondent 157	6	5	5	6	6	5	6	5	5	6	6
Respondent 158	5	6	6	5	4	6	5	6	6	5	5
Respondent 159	5	5	7	5	7	4	6	6	6	6	6
Respondent 160	5	5	5	5	6	7	4	7	7	4	4
Respondent 161	7	6	6	6	6	5	6	5	6	6	3

Respondent 162	5	6	6	5	4	6	5	6	6	5	5
Respondent 163	5	5	7	5	7	4	6	6	6	6	6
Respondent 164	5	5	5	5	6	7	4	7	7	4	4
Respondent 165	7	6	6	6	6	5	6	5	6	6	3
Respondent 166	5	5	4	5	5	5	5	7	6	5	5
Respondent 167	4	6	6	5	4	6	5	5	4	5	5
Respondent 168	5	4	5	5	7	4	6	5	6	6	6
Respondent 169	6	5	5	5	6	7	5	4	5	7	5
Respondent 170	6	5	4	6	6	5	5	5	5	5	5
Respondent 171	4	5	5	7	7	5	5	6	6	5	5
Respondent 172	6	6	6	5	6	6	6	4	5	4	6
Respondent 173	5	7	6	5	5	7	3	4	5	5	3
Respondent 174	5	5	4	5	5	5	6	6	5	6	6
Respondent 175	6	5	6	6	7	5	5	6	6	4	5
Respondent 176	5	4	5	7	5	7	4	5	4	5	4
Respondent 177	5	5	5	5	5	5	6	5	5	6	6
Respondent 178	5	6	6	5	7	6	5	5	6	6	5
Respondent 179	5	6	6	7	6	7	5	5	4	5	5
Respondent 180	5	5	7	5	7	7	4	5	4	5	4
Respondent 181	5	5	5	6	6	6	6	5	5	6	6
Respondent 182	7	6	6	7	5	7	5	5	6	6	5
Respondent 183	5	6	6	7	6	6	5	5	4	5	5
Respondent 184	5	5	7	6	7	7	4	6	6	5	4
Respondent 185	5	5	5	4	5	5	5	4	5	5	5
Respondent 186	7	6	6	7	5	7	6	5	5	5	6
Respondent 187	5	6	6	3	6	6	6	5	4	6	6
Respondent 188	5	5	7	4	7	5	4	5	5	4	4
Respondent 189	5	5	5	6	5	6	6	5	5	6	6
Respondent 190	7	6	6	7	6	6	5	5	6	6	5
Respondent 191	5	6	6	5	4	7	5	5	4	5	5
Respondent 192	5	5	7	7	5	5	4	6	6	5	4
Respondent 193	5	5	5	6	5	6	5	4	5	5	5
Respondent 194	7	6	6	6	6	6	6	5	5	5	6
Respondent 195	5	6	6	7	4	7	6	5	4	6	6
Respondent 196	5	5	7	6	6	5	4	5	5	4	4
Respondent 197	5	4	3	5	5	4	3	5	5	4	3
Respondent 198	6	5	5	5	6	5	5	5	6	5	5
Respondent 199	5	6	6	5	4	6	5	6	6	5	5
Respondent 200	5	5	7	5	7	4	6	6	6	6	6
Respondent 201	5	5	5	5	6	7	4	7	7	4	4
Respondent 202	7	6	6	6	6	5	6	5	6	6	3

Respondent 203	5	6	6	5	4	6	5	6	6	5	5
Respondent 204	5	5	7	5	7	4	6	6	6	6	6
Respondent 205	5	5	5	5	6	7	4	7	7	4	4
Respondent 206	7	6	6	6	6	5	6	5	6	6	3
Respondent 207	7	6	6	6	5	5	6	6	7	6	6
Respondent 208	4	5	5	4	5	4	5	3	7	6	6
Respondent 209	6	5	6	6	6	6	6	6	6	4	4
Respondent 210	5	6	5	7	5	7	5	1	4	6	5
Respondent 211	6	6	5	5	5	5	5	2	7	5	5
Respondent 212	4	5	6	5	6	5	6	6	7	5	4
Respondent 213	3	3	6	6	6	6	6	3	7	5	5
Respondent 214	1	4	5	7	5	7	5	6	6	5	6
Respondent 215	5	6	5	5	5	5	5	1	4	6	5
Respondent 216	6	6	6	5	6	5	6	2	7	5	5
Respondent 217	4	5	6	6	6	6	6	6	7	5	4
Respondent 218	7	6	6	5	6	5	6	3	7	6	6
Respondent 219	5	5	5	5	5	5	5	6	6	5	7
Respondent 220	6	5	5	4	5	4	5	1	4	5	5
Respondent 221	4	6	5	5	5	5	5	2	7	6	5
Respondent 222	7	6	5	6	5	6	5	6	7	6	6
Respondent 223	5	5	6	6	6	6	6	3	7	5	7
Respondent 224	6	5	4	4	4	4	4	6	6	5	5
Respondent 225	4	6	6	6	6	6	6	1	4	6	5
Respondent 226	6	6	6	5	6	5	6	2	7	6	6
Respondent 227	5	6	5	5	5	5	5	6	7	6	5
Respondent 228	6	6	5	4	5	4	5	3	7	5	5
Respondent 229	4	5	6	6	6	6	6	6	6	5	4
Respondent 230	3	3	5	7	5	7	5	1	4	5	5
Respondent 231	1	4	5	5	5	5	5	2	7	5	6
Respondent 232	2	2	6	5	6	5	6	6	7	6	6
Respondent 233	1	2	6	6	6	6	6	3	7	4	4
Respondent 234	5	6	5	7	5	7	5	6	6	6	6
Respondent 235	6	6	5	5	5	5	5	1	4	6	5
Respondent 236	4	5	6	5	6	5	6	2	7	5	5
Respondent 237	2	1	6	6	6	6	6	6	7	4	3
Respondent 238	1	2	6	5	6	5	6	3	7	3	4
Respondent 239	7	6	6	6	5	4	5	6	6	6	6
Respondent 240	5	6	6	5	4	6	5	6	6	5	5
Respondent 241	5	5	7	5	7	4	6	6	6	6	6
Respondent 242	5	5	5	5	6	7	4	7	7	4	4
Respondent 243	7	6	6	6	6	5	6	5	6	6	3

Respondent 244	5	4	5	4	5	4	5	4	5	4	5
Respondent 245	6	6	6	6	6	6	6	6	6	6	6
Respondent 246	5	7	5	7	5	7	5	7	5	7	5
Respondent 247	5	5	5	5	5	5	5	5	5	5	5
Respondent 248	6	5	6	5	6	5	6	5	6	5	6
Respondent 249	6	6	6	6	6	6	6	6	6	6	6
Respondent 250	6	5	5	6	6	5	4	5	5	4	4
Respondent 251	5	5	6	6	5	7	6	6	6	5	6
Respondent 252	5	5	4	5	5	5	5	7	6	5	5
Respondent 253	4	6	6	5	4	6	5	5	4	5	5
Respondent 254	5	4	5	5	7	4	6	5	6	6	6
Respondent 255	6	5	5	5	6	7	5	4	5	7	5
Respondent 256	6	5	4	6	6	5	5	5	5	5	5
Respondent 257	4	5	5	7	7	5	5	6	6	5	5
Respondent 258	6	6	6	5	6	6	6	4	5	4	6
Respondent 259	5	7	6	5	5	7	3	4	5	5	3
Respondent 260	5	5	4	5	5	5	6	6	5	6	6
Respondent 261	6	5	6	6	7	5	5	6	6	4	5
Respondent 262	5	4	5	7	5	7	4	5	4	5	4
Respondent 263	5	5	5	5	5	5	6	5	5	6	6
Respondent 264	5	6	6	5	7	6	5	5	6	6	5
Respondent 265	6	4	5	7	6	7	5	5	4	5	5
Respondent 266	3	4	5	5	7	7	4	5	4	5	4
Respondent 267	6	6	5	6	6	6	6	5	5	6	6
Respondent 268	5	6	6	7	5	7	5	5	6	6	5
Respondent 269	6	6	3	7	6	6	5	5	4	5	5
Respondent 270	4	5	6	6	7	7	4	6	6	5	4
Respondent 271	5	4	5	5	5	5	5	4	5	5	5
Respondent 272	7	6	6	7	5	7	6	5	5	5	6
Respondent 273	5	5	7	5	6	6	6	5	4	6	6
Respondent 274	6	5	5	6	7	5	4	5	5	4	4
Respondent 275	4	6	5	4	5	6	6	5	5	6	6
Respondent 276	7	5	4	7	6	6	5	5	6	6	5
Respondent 277	5	5	5	5	2	4	1	2	1	5	4
Respondent 278	5	5	6	5	2	2	3	2	3	2	3
Respondent 279	5	4	5	4	5	4	5	4	5	4	5
Respondent 280	6	6	6	6	6	6	6	6	6	6	6
Respondent 281	5	7	5	7	5	7	5	7	5	7	5
Respondent 282	5	5	5	5	5	5	5	5	5	5	5
Respondent 283	6	5	6	5	6	5	6	5	6	5	6
Respondent 284	6	6	6	6	6	6	6	6	6	6	6

Respondent 285	5	4	5	5	4	5	5	4	5	5	4
Respondent 286	7	6	6	7	6	6	7	6	6	7	6
Respondent 287	5	5	7	5	5	7	5	5	7	5	5
Respondent 288	6	5	5	6	5	5	6	5	5	6	5
Respondent 289	4	6	5	4	6	5	4	6	5	4	6
Respondent 290	7	5	4	7	5	4	7	5	4	7	5
Respondent 291	5	5	6	6	5	7	6	6	6	5	6
Respondent 292	5	5	4	5	5	5	5	7	6	5	5
Respondent 293	4	6	6	5	4	6	5	5	4	5	5
Respondent 294	5	4	5	5	7	4	6	5	6	6	6
Respondent 295	6	5	5	5	6	7	5	4	5	7	5
Respondent 296	6	5	4	6	6	5	5	5	5	5	5
Respondent 297	4	5	5	7	7	5	5	6	6	5	5
Respondent 298	6	6	6	5	6	6	6	4	5	4	6
Respondent 299	5	7	6	5	5	7	3	4	5	5	3
Respondent 300	5	5	4	5	5	5	6	6	5	6	6
Respondent 301	6	5	6	6	7	5	5	6	6	4	5
Respondent 302	5	4	5	7	5	7	4	5	4	5	4
Respondent 303	5	5	5	5	5	5	6	5	5	6	6
Respondent 304	5	6	6	5	7	6	5	5	6	6	5
Respondent 305	6	4	5	7	6	7	5	5	4	5	5
Respondent 306	3	4	5	5	7	7	4	5	4	5	4
Respondent 307	6	6	5	6	6	6	6	5	5	6	6
Respondent 308	5	6	6	7	5	7	5	5	6	6	5
Respondent 309	6	6	3	7	6	6	5	5	4	5	5
Respondent 310	4	5	6	6	5	4	5	5	4	5	4
Respondent 311	3	3	1	4	7	6	6	7	6	6	5
Respondent 312	1	4	2	7	5	5	7	5	5	7	6
Respondent 313	2	2	3	3	6	5	5	6	5	5	6
Respondent 314	1	2	2	4	4	6	5	4	6	5	4
Respondent 315	5	6	5	6	7	5	4	7	5	4	6
Respondent 316	6	6	6	7	5	5	5	5	5	5	5
Respondent 317	2	3	1	2	5	5	6	5	5	6	4
Respondent 318	3	4	2	2	6	6	4	6	6	4	2
Respondent 319	5	4	5	4	5	4	5	4	5	4	5
Respondent 320	6	6	6	6	6	6	6	6	6	6	6
Respondent 321	5	7	5	7	5	7	5	7	5	7	5
Respondent 322	5	5	5	5	5	5	5	5	5	5	5
Respondent 323	6	5	6	5	6	5	6	5	6	5	6
Respondent 324	6	6	6	6	6	6	6	6	6	6	6
Respondent 325	5	7	5	7	5	7	5	7	5	7	5

Respondent 326	5	6	6	5	6	6	5	5	6	6	5
Respondent 327	5	5	7	5	5	7	5	5	5	7	5
Respondent 328	6	5	5	6	5	5	6	6	5	5	6
Respondent 329	4	6	5	4	6	5	4	4	6	5	4
Respondent 330	5	5	4	5	5	4	5	5	5	4	5
Respondent 331	5	5	6	7	5	5	5	5	5	6	7
Respondent 332	4	3	5	5	7	5	5	7	5	5	4
Respondent 333	1	4	5	5	5	5	5	5	5	5	3
Respondent 334	3	2	7	6	6	7	6	6	7	6	5
Respondent 335	4	2	5	6	6	5	6	6	5	6	6
Respondent 336	2	3	5	5	7	5	5	7	5	5	4
Respondent 337	4	4	6	5	5	6	5	5	6	5	3
Respondent 338	3	1	4	6	5	4	6	5	4	6	5
Respondent 339	4	3	5	5	4	5	5	4	5	5	3
Respondent 340	6	5	5	5	6	7	5	5	5	5	4
Respondent 341	6	5	4	6	6	5	5	6	5	4	2
Respondent 342	4	5	5	7	7	5	5	4	6	5	5
Respondent 343	6	6	6	5	6	6	6	5	6	6	5
Respondent 344	6	5	5	5	6	7	5	7	6	5	2
Respondent 345	6	5	4	6	6	5	5	5	5	5	3
Respondent 346	4	5	5	7	7	5	5	6	5	4	5
Respondent 347	6	6	6	5	6	6	6	4	5	7	4
Respondent 348	6	5	5	5	6	7	5	7	5	6	3
Respondent 349	6	5	4	6	6	5	5	5	6	6	5
Respondent 350	4	5	5	7	7	5	5	5	7	7	5
Respondent 351	6	6	6	5	6	6	6	6	5	6	4
Respondent 352	6	5	5	5	6	7	6	5	6	6	5
Respondent 353	6	5	4	6	6	5	7	5	5	7	5
Respondent 354	4	5	5	7	7	5	5	6	5	5	6
Respondent 355	6	6	6	5	6	6	5	4	6	5	4
Respondent 356	6	5	5	5	6	7	4	5	5	4	5
Respondent 357	6	5	4	6	6	5	6	7	5	6	7
Respondent 358	4	5	5	7	7	5	6	5	5	6	5
Respondent 359	6	6	6	5	6	6	7	5	5	7	5
Respondent 360	6	5	5	5	6	7	6	6	6	6	6
Respondent 361	5	6	6	5	6	6	6	7	5	6	7
Respondent 362	5	5	7	5	5	7	6	5	5	6	5
Respondent 363	6	5	5	6	5	5	6	5	6	6	5
Respondent 364	4	6	5	4	6	5	7	5	5	7	5
Respondent 365	5	5	4	5	5	4	5	5	5	4	5
Respondent 366	5	5	6	7	5	5	5	5	5	6	7

Respondent 367	5	6	6	5	6	6	5	5	6	6	5
Respondent 368	5	5	7	5	5	7	5	5	5	7	5
Respondent 369	6	5	5	6	5	5	6	6	5	5	6
Respondent 370	4	6	5	4	6	5	4	4	6	5	4
Respondent 371	5	5	4	5	5	4	5	5	5	4	5
Respondent 372	4	6	6	5	4	6	5	5	4	5	5
Respondent 373	5	4	5	5	7	4	6	5	6	6	6
Respondent 374	6	5	5	5	6	7	5	4	5	7	5
Respondent 375	6	5	4	6	6	5	5	5	5	5	5
Respondent 376	4	5	5	7	7	5	5	6	6	5	5
Respondent 377	6	6	6	5	6	6	6	4	5	4	6
Respondent 378	5	7	6	5	5	7	3	4	5	5	3
Respondent 379	5	6	6	5	6	6	5	5	6	6	5
Respondent 380	5	5	7	5	5	7	5	5	5	7	5
Respondent 381	6	5	5	6	5	5	6	6	5	5	6
Respondent 382	4	6	5	4	6	5	4	4	6	5	4
Respondent 383	5	5	4	5	5	4	5	5	5	4	5

Response to questionnaire (36-43)

	Eitem1	Eitem2	Eitem3	Eitem4	Eitem5	Eitem6	Eitem7	Eitem8
Respondent 1	7	6	7	2	7	5	4	7
Respondent 2	5	4	5	2	5	7	5	5
Respondent 3	7	5	6	4	7	7	6	6
Respondent 4	6	5	7	2	6	5	4	7
Respondent 5	6	6	5	2	6	7	5	5
Respondent 6	7	6	5	6	7	6	5	4
Respondent 7	5	4	6	6	5	6	6	5
Respondent 8	7	6	4	7	7	7	6	5
Respondent 9	5	5	5	6	5	5	4	5
Respondent 10	6	5	6	6	6	7	6	6
Respondent 11	7	6	4	5	7	5	5	7
Respondent 12	5	4	6	5	5	6	5	5
Respondent 13	7	5	6	4	7	7	6	6
Respondent 14	7	6	7	2	7	5	4	7
Respondent 15	5	4	5	2	5	7	5	5
Respondent 16	7	5	6	4	7	7	6	6
Respondent 17	7	6	7	2	7	5	4	7
Respondent 18	5	4	5	2	5	7	5	5
Respondent 19	7	5	6	4	7	7	6	6
Respondent 20	5	6	7	2	5	5	4	7
Respondent 21	7	6	5	2	7	7	5	5
Respondent 22	5	5	5	5	5	5	6	5
Respondent 23	5	6	6	5	7	6	5	5
Respondent 24	6	4	5	7	6	7	5	5
Respondent 25	3	4	5	5	7	7	4	5
Respondent 26	6	6	5	6	6	6	6	5
Respondent 27	5	6	6	7	5	7	5	5
Respondent 28	6	6	3	7	6	6	5	5
Respondent 29	4	5	6	6	7	7	4	6
Respondent 30	3	3	1	4	5	5	5	4
Respondent 31	1	4	2	7	5	7	6	5
Respondent 32	2	2	3	3	6	6	6	5
Respondent 33	5	4	7	2	1	5	4	7
Respondent 34	7	5	5	2	2	7	5	5
Respondent 35	7	6	6	4	4	7	6	6
Respondent 36	5	4	7	2	1	5	4	7
Respondent 37	7	5	5	2	2	7	5	5
Respondent 38	5	5	5	5	5	5	6	5

Respondent 39	5	2	5	2	5	2	5	2
Respondent 40	6	4	6	4	6	4	6	4
Respondent 41	7	2	7	2	7	2	7	2
Respondent 42	5	2	5	2	5	2	5	2
Respondent 43	5	6	5	6	5	6	5	6
Respondent 44	6	6	6	6	6	6	6	6
Respondent 45	4	7	4	7	4	7	4	7
Respondent 46	5	2	2	7	5	5	4	2
Respondent 47	5	5	6	6	5	7	6	6
Respondent 48	5	5	4	5	5	5	5	7
Respondent 49	4	6	6	5	4	6	5	5
Respondent 50	7	6	6	4	4	7	6	6
Respondent 51	5	4	7	2	1	5	4	7
Respondent 52	7	5	5	2	2	7	5	5
Respondent 53	7	6	6	4	4	7	6	6
Respondent 54	5	4	7	2	1	5	4	7
Respondent 55	7	5	5	2	2	7	5	5
Respondent 56	7	6	6	4	4	7	6	6
Respondent 57	5	4	7	2	1	5	4	7
Respondent 58	7	5	5	2	2	7	5	5
Respondent 59	5	5	5	5	5	5	6	5
Respondent 60	5	6	6	5	7	6	5	5
Respondent 61	6	4	5	7	6	7	5	5
Respondent 62	3	4	5	5	7	7	4	5
Respondent 63	6	6	5	6	6	6	6	5
Respondent 64	5	6	6	7	5	7	5	5
Respondent 65	6	6	3	7	6	6	5	5
Respondent 66	4	5	6	6	7	7	4	6
Respondent 67	3	3	1	4	5	5	5	4
Respondent 68	1	4	2	7	5	7	6	5
Respondent 69	2	2	3	3	6	6	6	5
Respondent 70	5	6	6	5	7	6	5	5
Respondent 71	6	4	5	7	6	7	5	5
Respondent 72	3	4	5	5	7	7	4	5
Respondent 73	6	6	5	6	6	6	6	5
Respondent 74	5	6	6	7	5	7	5	5
Respondent 75	5	2	2	7	5	5	4	2
Respondent 76	5	2	5	2	5	2	5	2
Respondent 77	6	4	6	4	6	4	6	4
Respondent 78	7	2	7	2	7	2	7	2
Respondent 79	5	2	5	2	5	2	5	2

Respondent 80	5	2	5	2	5	2	5	2
Respondent 81	6	4	6	4	6	4	6	4
Respondent 82	7	2	7	2	7	2	7	2
Respondent 83	5	2	5	2	5	2	5	2
Respondent 84	5	6	5	6	5	6	5	6
Respondent 85	5	5	4	5	5	5	5	7
Respondent 86	5	4	7	2	1	5	4	7
Respondent 87	7	5	5	2	2	7	5	5
Respondent 88	7	6	6	4	4	7	6	6
Respondent 89	5	4	7	2	1	5	4	7
Respondent 90	5	6	5	6	5	6	5	4
Respondent 91	6	6	6	6	6	6	6	5
Respondent 92	4	5	4	7	4	7	6	5
Respondent 93	6	5	5	6	6	5	4	5
Respondent 94	5	5	5	2	5	2	5	6
Respondent 95	5	2	6	4	6	4	6	2
Respondent 96	6	4	7	2	7	2	7	2
Respondent 97	7	2	5	2	5	2	5	4
Respondent 98	5	2	5	6	5	6	5	2
Respondent 99	5	2	6	6	6	6	6	2
Respondent 100	7	6	4	7	4	7	4	6
Respondent 101	5	4	5	6	5	6	5	7
Respondent 102	7	5	6	6	6	6	6	5
Respondent 103	7	6	4	5	4	5	4	6
Respondent 104	5	4	6	5	6	5	6	7
Respondent 105	7	5	6	4	6	4	6	5
Respondent 106	5	5	7	2	7	2	7	5
Respondent 107	5	6	5	2	5	2	5	5
Respondent 108	6	4	6	4	6	4	6	5
Respondent 109	3	4	7	2	7	2	7	5
Respondent 110	6	6	5	2	5	2	5	5
Respondent 111	5	6	6	4	6	4	6	5
Respondent 112	6	6	7	2	7	2	7	5
Respondent 113	4	5	5	2	5	2	5	6
Respondent 114	3	3	5	5	5	5	5	4
Respondent 115	5	2	5	2	5	2	5	2
Respondent 116	6	4	6	4	6	4	6	4
Respondent 117	7	2	7	2	7	2	7	2
Respondent 118	5	2	5	2	5	2	5	2
Respondent 119	5	6	5	6	5	6	5	6
Respondent 120	6	6	6	6	6	6	6	6

Respondent 121	5	2	5	2	5	2	5	2
Respondent 122	6	4	6	4	6	4	6	4
Respondent 123	7	2	7	2	7	2	7	2
Respondent 124	5	2	5	2	5	2	5	2
Respondent 125	5	6	5	6	5	6	5	6
Respondent 126	6	6	6	6	6	6	6	6
Respondent 127	4	7	4	7	4	7	4	7
Respondent 128	5	6	5	6	5	6	5	6
Respondent 129	6	6	6	6	6	6	6	6
Respondent 130	6	6	5	6	5	7	4	7
Respondent 131	7	7	5	5	5	5	5	5
Respondent 132	5	6	6	5	4	6	5	6
Respondent 133	5	5	7	5	7	4	6	6
Respondent 134	5	5	5	5	6	7	4	7
Respondent 135	7	6	6	6	6	5	6	5
Respondent 136	6	5	6	7	7	5	5	7
Respondent 137	7	6	6	5	6	6	5	5
Respondent 138	5	4	7	5	5	7	4	6
Respondent 139	7	5	5	5	5	5	7	4
Respondent 140	6	5	6	7	6	6	6	7
Respondent 141	6	6	6	5	6	6	6	5
Respondent 142	7	4	7	5	5	7	7	5
Respondent 143	6	6	5	6	5	5	6	6
Respondent 144	6	5	7	4	6	5	5	7
Respondent 145	5	4	3	5	5	4	5	5
Respondent 146	6	5	5	5	6	5	6	6
Respondent 147	4	4	5	6	4	4	5	6
Respondent 148	5	3	6	4	5	3	6	6
Respondent 149	5	2	5	2	5	2	5	2
Respondent 150	6	4	6	4	6	4	6	4
Respondent 151	7	2	7	2	7	2	7	2
Respondent 152	5	2	5	2	5	2	5	2
Respondent 153	5	6	5	6	5	6	5	6
Respondent 154	6	6	6	6	6	6	6	6
Respondent 155	4	7	4	7	4	7	4	7
Respondent 156	5	6	5	6	5	6	5	6
Respondent 157	6	6	6	6	6	6	6	6
Respondent 158	4	5	4	5	4	5	4	5
Respondent 159	6	5	6	5	6	5	6	5
Respondent 160	6	4	6	4	6	4	6	4
Respondent 161	7	2	7	2	7	2	7	2

Respondent 162	5	6	6	5	4	6	5	6
Respondent 163	5	5	7	5	7	4	6	6
Respondent 164	5	5	5	5	6	7	4	7
Respondent 165	7	6	6	6	6	5	6	5
Respondent 166	5	5	4	5	5	5	5	7
Respondent 167	4	6	6	5	4	6	5	5
Respondent 168	5	4	5	5	7	4	6	5
Respondent 169	6	5	5	5	6	7	5	4
Respondent 170	6	5	4	6	6	5	5	5
Respondent 171	4	5	5	7	7	5	5	6
Respondent 172	6	6	6	5	6	6	6	4
Respondent 173	5	7	6	5	5	7	3	4
Respondent 174	5	5	4	5	5	5	6	6
Respondent 175	6	5	6	6	7	5	5	6
Respondent 176	5	4	5	7	5	7	4	5
Respondent 177	5	5	5	5	5	5	6	5
Respondent 178	5	6	6	5	7	6	5	5
Respondent 179	5	6	6	7	6	7	5	5
Respondent 180	5	5	7	5	7	7	4	5
Respondent 181	5	5	5	6	6	6	6	5
Respondent 182	7	6	6	7	5	7	5	5
Respondent 183	5	6	6	7	6	6	5	5
Respondent 184	5	5	7	6	7	7	4	6
Respondent 185	5	5	5	4	5	5	5	4
Respondent 186	7	6	6	7	5	7	6	5
Respondent 187	5	6	6	3	6	6	6	5
Respondent 188	5	5	7	4	7	5	4	5
Respondent 189	5	5	5	6	5	6	6	5
Respondent 190	7	6	6	7	6	6	5	5
Respondent 191	5	6	6	5	4	7	5	5
Respondent 192	5	5	7	7	5	5	4	6
Respondent 193	5	5	5	6	5	6	5	4
Respondent 194	7	6	6	6	6	6	6	5
Respondent 195	5	6	6	7	4	7	6	5
Respondent 196	5	5	7	6	6	5	4	5
Respondent 197	5	4	3	5	5	4	3	5
Respondent 198	6	5	5	5	6	5	5	5
Respondent 199	5	6	6	5	4	6	5	6
Respondent 200	5	5	7	5	7	4	6	6
Respondent 201	5	5	5	5	6	7	4	7
Respondent 202	7	6	6	6	6	5	6	5

Respondent 203	5	2	5	2	5	2	5	2
Respondent 204	6	4	6	4	6	4	6	4
Respondent 205	7	2	7	2	7	2	7	2
Respondent 206	5	2	5	2	5	2	5	2
Respondent 207	5	6	5	6	5	6	5	6
Respondent 208	6	6	6	6	6	6	6	6
Respondent 209	4	7	4	7	4	7	4	7
Respondent 210	5	6	5	7	5	7	5	1
Respondent 211	6	6	5	5	5	5	5	2
Respondent 212	4	5	6	5	6	5	6	6
Respondent 213	3	3	6	6	6	6	6	3
Respondent 214	1	4	5	7	5	7	5	6
Respondent 215	5	6	5	5	5	5	5	1
Respondent 216	6	6	6	5	6	5	6	2
Respondent 217	4	5	6	6	6	6	6	6
Respondent 218	7	6	6	5	6	5	6	3
Respondent 219	5	5	5	5	5	5	5	6
Respondent 220	6	5	5	4	5	4	5	1
Respondent 221	4	6	5	5	5	5	5	2
Respondent 222	7	6	5	6	5	6	5	6
Respondent 223	5	5	6	6	6	6	6	3
Respondent 224	6	5	4	4	4	4	4	6
Respondent 225	5	6	6	6	6	6	6	1
Respondent 226	6	6	6	5	6	5	6	2
Respondent 227	7	5	5	5	5	5	5	6
Respondent 228	5	5	5	4	5	4	5	3
Respondent 229	5	6	6	6	6	6	6	6
Respondent 230	6	6	5	7	5	7	5	1
Respondent 231	5	6	5	5	5	5	5	2
Respondent 232	6	5	6	5	6	5	6	6
Respondent 233	5	4	6	6	6	6	6	3
Respondent 234	5	6	5	7	5	7	5	6
Respondent 235	6	6	5	5	5	5	5	1
Respondent 236	7	5	6	5	6	5	6	2
Respondent 237	5	5	6	6	6	6	6	6
Respondent 238	5	6	6	5	6	5	6	3
Respondent 239	6	6	6	6	5	4	5	6
Respondent 240	5	6	6	5	4	6	5	6
Respondent 241	6	5	7	5	7	4	6	6
Respondent 242	5	4	5	5	6	7	4	7
Respondent 243	5	6	6	6	6	5	6	5

Respondent 244	5	6	5	6	5	6	5	6
Respondent 245	6	6	6	6	6	6	6	6
Respondent 246	7	5	7	5	7	5	7	5
Respondent 247	5	5	5	5	5	5	5	5
Respondent 248	5	6	5	6	5	6	5	6
Respondent 249	6	6	6	6	6	6	6	6
Respondent 250	6	5	5	6	6	5	4	5
Respondent 251	5	5	6	6	5	7	6	6
Respondent 252	5	5	4	5	5	5	5	7
Respondent 253	4	6	6	5	4	6	5	5
Respondent 254	5	4	5	5	7	4	6	5
Respondent 255	6	5	5	5	6	7	5	4
Respondent 256	6	5	4	6	6	5	5	5
Respondent 257	4	5	5	7	7	5	5	6
Respondent 258	6	6	6	5	6	6	6	4
Respondent 259	5	7	6	5	5	7	3	4
Respondent 260	5	5	4	5	5	5	6	6
Respondent 261	6	5	6	6	7	5	5	6
Respondent 262	5	4	5	7	5	7	4	5
Respondent 263	5	5	5	5	5	5	6	5
Respondent 264	5	6	6	5	7	6	5	5
Respondent 265	6	4	5	7	6	7	5	5
Respondent 266	3	4	5	5	7	7	4	5
Respondent 267	6	6	5	6	6	6	6	5
Respondent 268	5	6	6	7	5	7	5	5
Respondent 269	6	6	3	7	6	6	5	5
Respondent 270	4	5	6	6	7	7	4	6
Respondent 271	5	4	5	5	5	5	5	4
Respondent 272	7	6	6	7	5	7	6	5
Respondent 273	5	5	7	5	6	6	6	5
Respondent 274	6	5	5	6	7	5	4	5
Respondent 275	4	6	5	4	5	6	6	5
Respondent 276	7	5	4	7	6	6	5	5
Respondent 277	5	5	5	5	2	4	1	2
Respondent 278	5	5	6	5	2	2	3	2
Respondent 279	5	4	5	4	5	4	5	4
Respondent 280	6	6	6	6	6	6	6	6
Respondent 281	5	6	5	6	5	6	5	6
Respondent 282	6	6	6	6	6	6	6	6
Respondent 283	7	5	7	5	7	5	7	5
Respondent 284	5	5	5	5	5	5	5	5

Respondent 285	6	5	7	6	6	5	7	6
Respondent 286	5	5	5	5	5	5	5	5
Respondent 287	5	4	6	5	5	4	6	5
Respondent 288	5	7	4	6	5	7	4	6
Respondent 289	5	6	7	5	5	6	7	5
Respondent 290	6	6	5	5	6	6	5	5
Respondent 291	5	5	6	6	5	7	6	6
Respondent 292	5	5	4	5	5	5	5	7
Respondent 293	4	6	6	5	4	6	5	5
Respondent 294	5	4	5	5	7	4	6	5
Respondent 295	6	5	5	5	6	7	5	4
Respondent 296	6	5	4	6	6	5	5	5
Respondent 297	4	5	5	7	7	5	5	6
Respondent 298	6	6	6	5	6	6	6	4
Respondent 299	5	7	6	5	5	7	3	4
Respondent 300	5	5	4	5	5	5	6	6
Respondent 301	6	5	6	6	7	5	5	6
Respondent 302	5	4	5	7	5	7	4	5
Respondent 303	5	5	5	5	5	5	6	5
Respondent 304	5	6	6	5	7	6	5	5
Respondent 305	6	4	5	7	6	7	5	5
Respondent 306	3	4	5	5	7	7	4	5
Respondent 307	6	6	5	6	6	6	6	5
Respondent 308	5	6	6	7	5	7	5	5
Respondent 309	6	6	3	7	6	6	5	5
Respondent 310	4	5	6	6	5	4	5	5
Respondent 311	3	3	1	4	7	6	6	7
Respondent 312	1	4	2	7	5	5	7	5
Respondent 313	2	2	3	3	6	5	5	6
Respondent 314	1	2	2	4	4	6	5	4
Respondent 315	5	6	5	6	7	5	4	7
Respondent 316	6	6	6	7	5	5	5	5
Respondent 317	2	3	1	2	5	5	6	5
Respondent 318	3	4	2	2	6	6	4	6
Respondent 319	5	4	5	4	5	4	5	4
Respondent 320	6	6	6	6	6	6	6	6
Respondent 321	5	7	5	7	5	7	5	7
Respondent 322	5	5	5	5	5	5	5	5
Respondent 323	6	5	6	5	6	5	6	5
Respondent 324	6	6	6	6	6	6	6	6
Respondent 325	5	7	5	7	5	7	5	7

Respondent 326	6	5	7	6	6	5	7	6
Respondent 327	5	5	5	5	5	5	5	5
Respondent 328	5	4	6	5	5	4	6	5
Respondent 329	5	7	4	6	5	7	4	6
Respondent 330	5	6	7	5	5	6	7	5
Respondent 331	6	6	5	5	6	6	5	5
Respondent 332	6	5	6	6	5	6	6	7
Respondent 333	7	5	5	7	5	5	7	5
Respondent 334	5	6	5	5	6	5	5	6
Respondent 335	5	4	6	5	4	6	5	6
Respondent 336	4	5	5	4	5	5	4	7
Respondent 337	6	7	5	6	7	5	6	5
Respondent 338	6	5	5	6	5	5	6	5
Respondent 339	7	5	5	7	5	5	7	4
Respondent 340	6	6	6	6	6	6	6	5
Respondent 341	6	7	5	6	7	5	6	6
Respondent 342	6	5	5	6	5	5	6	4
Respondent 343	6	5	6	6	5	6	6	5
Respondent 344	7	5	5	7	5	5	7	7
Respondent 345	5	6	5	5	6	5	5	5
Respondent 346	5	4	6	5	4	6	5	6
Respondent 347	4	5	5	4	5	5	4	4
Respondent 348	6	7	5	6	7	5	6	7
Respondent 349	6	5	4	6	6	5	5	5
Respondent 350	4	5	5	7	7	5	5	5
Respondent 351	6	6	6	5	6	6	6	6
Respondent 352	6	5	5	5	6	7	5	7
Respondent 353	6	5	4	6	6	5	5	2
Respondent 354	4	5	5	7	7	5	5	3
Respondent 355	6	6	6	5	6	6	6	4
Respondent 356	6	5	5	5	6	7	5	1
Respondent 357	6	5	4	6	6	5	5	3
Respondent 358	4	5	5	7	7	5	5	4
Respondent 359	6	6	6	5	6	6	6	2
Respondent 360	6	5	5	5	6	7	5	2
Respondent 361	5	6	6	5	6	6	5	5
Respondent 362	6	5	6	6	5	6	6	5
Respondent 363	7	5	5	7	5	5	7	5
Respondent 364	5	6	5	5	6	5	5	6
Respondent 365	5	4	6	5	4	6	5	4
Respondent 366	4	5	5	4	5	5	4	5

Respondent 367	6	5	6	6	5	6	6	5
Respondent 368	7	5	5	7	5	5	7	5
Respondent 369	5	6	5	5	6	5	5	6
Respondent 370	5	4	6	5	4	6	5	4
Respondent 371	5	5	4	5	5	4	5	5
Respondent 372	4	6	6	5	4	6	5	5
Respondent 373	5	4	5	5	7	4	6	5
Respondent 374	6	5	5	5	6	7	5	4
Respondent 375	6	5	4	6	6	5	5	5
Respondent 376	4	5	5	7	7	5	5	6
Respondent 377	6	6	6	5	6	6	6	4
Respondent 378	5	7	6	5	5	7	3	4
Respondent 379	5	6	6	5	6	6	5	5
Respondent 380	6	5	6	6	5	6	6	5
Respondent 381	7	5	5	7	5	5	7	5
Respondent 382	5	6	5	5	6	5	5	6
Respondent 383	5	4	6	5	4	6	5	4
